# Detection of Novel QTLs for Late Blight Resistance Derived from the Wild Potato Species *Solanum microdontum* and *Solanum pampasense*

**DOI:** 10.3390/genes11070732

**Published:** 2020-06-30

**Authors:** Fergus Meade, Ronald Hutten, Silke Wagener, Vanessa Prigge, Emmet Dalton, Hanne Grethe Kirk, Denis Griffin, Dan Milbourne

**Affiliations:** 1 Teagasc, Crop Science Department, Oak Park, R93 XE12 Carlow, Ireland; fergus.meade@teagasc.ie (F.M.); denis.griffin@teagasc.ie (D.G.); 2 Wageningen University & Research (WUR), 6708 PB Wageningen, The Netherlands; ronald.hutten@wur.nl; 3 SaKa Pflanzenzucht GmbH & Co., 22761 Hamburg, Germany; silke.wagener@saka-pflanzenzucht.de (S.W.); vanessa.prigge@saka-pflanzenzucht.de (V.P.); 4 Aardevo, 8308 PB Nagele, The Netherlands; emmet.dalton@aardevo.com; 5 Danespo, 7323 Give, Denmark; hgk@danespo.dk

**Keywords:** potato, breeding, late blight, *Phytophthora infestans*, marker-assisted selection, QTL

## Abstract

Wild potato species continue to be a rich source of genes for resistance to late blight in potato breeding. Whilst many dominant resistance genes from such sources have been characterised and used in breeding, quantitative resistance also offers potential for breeding when the loci underlying the resistance can be identified and tagged using molecular markers. In this study, F_1_ populations were created from crosses between blight susceptible parents and lines exhibiting strong partial resistance to late blight derived from the South American wild species *Solanum microdontum* and *Solanum pampasense*. Both populations exhibited continuous variation for resistance to late blight over multiple field-testing seasons. High density genetic maps were created using single nucleotide polymorphism (SNP) markers, enabling mapping of quantitative trait loci (QTLs) for late blight resistance that were consistently expressed over multiple years in both populations. In the population created with the *S. microdontum* source, QTLs for resistance consistently expressed over three years and explaining a large portion (21–47%) of the phenotypic variation were found on chromosomes 5 and 6, and a further resistance QTL on chromosome 10, apparently related to foliar development, was discovered in 2016 only. In the population created with the *S. pampasense* source, QTLs for resistance were found in over two years on chromosomes 11 and 12. For all loci detected consistently across years, the QTLs span known R gene clusters and so they likely represent novel late blight resistance genes. Simple genetic models following the effect of the presence or absence of SNPs associated with consistently effective loci in both populations demonstrated that marker assisted selection (MAS) strategies to introgress and pyramid these loci have potential in resistance breeding strategies.

## 1. Introduction

Late blight, caused by the oomycete pathogen *Phytophthora infestans*, continues to present a challenge for potato production in temperate regions worldwide, and breeding for resistance is to be an important breeding objective. During the course of a breeding initiative to introgress resistance from wild species sources into an elite cultivated potato background we observed that resistance sourced originally from specific accessions of the wild potato species *Solanum microdontum* and *Solanum pampasense* exhibit quantitative inheritance patterns. Whilst, in our experience, quantitative disease and pest resistance loci are useful tools in the development of varietal resistance, the availability of genetic markers to efficiently track their introgression and enable strategies such as pyramiding multiple partial resistances to achieve a more complete resistance phenotype. In this study we report the identification of quantitative trait loci (QTL) underlying resistance in these sources, and validation of the use of linked polymorphisms for marker assisted selection (MAS)-driven pyramiding approaches in breeding.

*S. microdontum* is found in Bolivia and Argentina [1] and accessions with resistance to late blight have been described in Argentinian [2], German [3], Russian [4] and US [5] collections. Its potential for use as a source of late blight resistance in potato breeding programmes has been well documented [6,7,8,9]. Dominant gene action was observed in crosses with *S. microdontum* sources and susceptible diploid genotypes of *Solanum. tuberosum* [7] and resistance was associated with a strong hypersensitive reaction [6]. Resistance from MCD167 was explained by a large QTL on chr04 [10]. The same study described a major resistance QTL from MCD178 on chr10. QTLs were also detected on chr05 but not consistently in all populations. Using a population developed from BGRC 27353/CGN20640, a major QTL associated with foliar late blight resistance was described [11]. Although it could not be mapped to a specific chromosome it was deemed to be different to that described by Sandbrink et al. [10]. The *Rpi-mcd1* locus was mapped to a cluster of Nucleotide Binding-Leucine Rich Repeat (NB-LRR) genes on chr04 [12], coinciding with a NB-LRR gene cluster in which *R2*, *R2-like*, *Rpi-blb3* and *Rpi-abpt* have been mapped. The authors concluded that *Rpi-mcd1* is a weak but broad-spectrum resistance gene. In addition to NB-LRR based resistance genes, *S. microdontum* has also been shown to be a potential source of broad spectrum non-NB-LRR-based resistance. Du et al. [13] demonstrated that the receptor-like protein elicitin response (ELR) from *S. microdontum* mediates extracellular recognition of the elicitin domain, a molecular pattern that is conserved in *Phytophthora* species. Recognition of pathogen elicitin is mediated by ELR cell surface receptors in *S. microdontum* which triggers activation of immune signalling and a sustained reactive oxygen species burst that results in cell death. Transfer of ELR from *S. microdontum* to *S. tuberosum* resulted in broad spectrum resistance to late blight. Additionally, late blight resistant *S. microdontum* sources can be used in breeding for increased calcium content of tubers. 

*S. pampasense* has been much less utilised in resistance breeding to date. The species has its origin in Southern Peru and is thought to be a hybrid of *Solanum bukasovii* and *Solanum marinasense* [14]. Accessions of this species from the Commonwealth Potato Collection have reported resistance to potato cyst nematotodes [15]. Accessions of *S. pampasense* were recently shown to be susceptible to *Globodera pallida* but no data was available for late blight resistance [3].

In this study, previously uncharacterised sources of late blight derived from *S. microdontum* and *S. pampasense* were subject to genetic mapping to identify the loci underlying their resistance to *P. infestans*. Breeding populations were established with each of the resistant sources. Significantly, both populations exhibited quantitative rather than qualitative resistance. Dense, single nucleotide polymorphism (SNP)-based linkage maps were generated for both populations and multiple QTLs were discovered using phenotypic data recorded over two or three years. Loci exhibiting a consistent effect on resistance across years were discovered in both cases, and their potential for use in MAS-based breeding strategies was investigated.

## 2. Materials and Methods

### 2.1. Mapping Populations

The *S. microdontum* source, MCD 360-8, is derived from collection number OKA 4478 (by A. Okado in Argentina [16]). Clone IVP07-174-17 was produced from a cross between MCD 360-8 and HOS 01-8036, which was subsequently used in a cross with RH91-237-6 to produce the resistant parent of the MCD population described in this study, IVP10-281-1. A diploid mapping population was developed from a cross between blight susceptible diploid breeding clone, IVP06-158-2 (female) and IVP10-281-1 (male). 

The *S. pampasense* source, PAM 287-2, is derived from CPC 7328 (unknown origin). Clone RH06-862-2 was produced by crossing this source with RH02-192-5, which was then crossed with the commercial cultivar MIRANDA to produce RH4X-753-3, the female resistant parent of the PAM population comprising 71 genotypes. RH4X-753-3 was independently crossed with the male parents DIVAA, IVP4X-132-6, MERIDA and VOYAGER, producing four subpopulations comprising 19, 20, 21 and 11 genotypes, respectively.

### 2.2. Resistance Screening and Phenotypic Data Collection

The MCD population was evaluated for resistance to late blight over four consecutive years at trial sites in Hohenlieth, Germany. Planting took place on 6 May 2016, 15 May 2017 and 17 April 2019. Artificial inoculation was carried out with a mixture of *P. infestans* (pathotypes 1, 2, 3, 4, 5, 6, 7, 8, 9, 10, 11) obtained from the Max-Planck Institute for Plant Breeding Research, Cologne, Germany. This mixture is tested each year by Solana Research to have all pathotypes still present. Immediately prior to evening inoculations trials were sprinkler irrigated for 15 min. Inoculations were carried out on 30 June 2016 (900 zoospores per mL), 13 July 2017 (2200 zoospores per mL) and 20 July 2019 (600 zoospores per mL). Trials were irrigated with a sprinkler system morning and evening for approximately 1 h during infection establishment, for as long as lesions were visible in susceptible checks. Genotypes were scored on six dates twice a week from 11 to 29 July 2016, seven dates twice a week from 7 July to 8 August 2017 and ten dates twice a week from 4 July to 5 August 2019. In 2018, a trial was established but due to the particularly dry summer experienced in Europe no blight infection took hold. The lattice-adjusted mean area under the disease progress curve (AUDPC) was calculated for each genotype across two reps before relative AUDPC (AUDPCr) was computed relative to the mean of the check varieties—ALMONDA, CAMPINA, CAROLUS and CONNECT in 2016; ALMONDA, CAMPINA and ATHLETE in 2017; ATHLETE, BURANA, CAMPINA, CONNECT, INNOVATOR, SOLIST and TWISTER in 2019. In 2016 the full set of 101 F1 clones was assessed. Two tubers per genotype were sown, there was no replication and the trial was not randomised. Of these, 43 derived from tubers harvested from the field in the previous year (“Field subset”) and 58 derived from tubers obtained from plants grown in pots (“Pot subset”). Emergence was scored (1 = not emerged to 9 = fully emerged) because of an anticipated developmental difference between the subgroups. The 2017 trial had a randomised lattice design with two reps of four tubers harvested from the Pot subset from the previous year. In addition to late blight resistance, foliar maturity and development trait data were also collected [17]. Maturity determines the earliness or lateness of senescence for a clone while development evaluates the amount of foliage produced (1 = plants have not yet emerged, 9 = very well developed plants). Finally, in 2019, 51 genotypes (from the Pot subset were assessed, with two reps of four tubers per genotype and a randomised lattice design was followed. Maturity and development were scored in addition to AUDPCr. 

Single plots containing four plants per genotype were planted in 2016 (May 2nd) and 2017 (May 4th) growing seasons in Wageningen, the Netherlands. Innoculation with *P. infestans* isolate IPO-C (race 1, 2, 3, 4, 5, 6, 7, 10, 11) took place after 9 p.m. on 1 July 2016 and 3 July 2017. The inocolum was produced on leaves (8 per 100 m^2^) of variety BINTJE placed in a climate chamber at 18 °C for 6 days. Leaves were rinsed with water at 4 °C and 6–8 batches of inoculum in 5 L batches were prepared, which ended up together in the tank of the spraying device. On the day of inoculation, the field was irrigated for 1 h in the morning and for 15 min just before inoculation. The first day after inoculation there was no irrigation, but from then irrigation took place daily for 15 min at 7 a.m. and 8 p.m. Genotypes were scored on a plot basis (no replicates) once per week on four occasions each during the month of July in both 2016 (July 1st–25th) and 2017 (July 3rd–25th). The AUDPC for each plot was calculated. 

### 2.3. DNA Extraction

Genomic DNA was extracted from leaf tissue of genotypes from both populations using a cetyl trimethylammonium bromide (CTAB) protocol. Leaf material from the 2016 trials was collected in 2 mL eppendorfs and freeze dried for 72 h prior to mechanical disruption with a glass bead in a Tissue Lyser. 1 mL of 0.8% CTAB-β-mercaptoethanol was added to the ground tissue and incubated for 1 h at 65 °C. Samples were spun at 13,000 rpm for 10 min. A total of 900 μL was taken from each tube and added to a fresh 2 mL tube. 1:1 phenol:chloroform IAA (25:24:1) was added and vortexed to mix. Following centrifugation at 13,000 rpm for 5 min, 700 μL was removed and added to a fresh 2 mL tube with 1:1 phenol:chloroform IAA (25:24:1). The sample was again vortexed to mix prior to centrifugation at 13,000 rpm for 5 min. 550 μL was then removed and added to a fresh 1.5 mL Eppendorf with 1:1 prechilled isopropanol. DNA precipitation followed at −20 °C for 2 h before centrifugation at 13,000 rpm for 10 min. The DNA pellet formed was cleaned by rinsing with 500 μL 70% ethanol. Samples were incubated on ice for 10 min prior to a further spin at 13,000 rpm for 5 min. The ethanol was removed and samples were left to air dry before resuspension with 100 μL TE 0.1 mM EDTA. Finally, samples were treated with RNaseA. DNA was quantified using the Quant-iT PicoGreen dsDNA assay (Invitrogen, Carlsbad, CA, USA) as per the manufacturer’s instructions. Fluorescence was measured using a BioTek (Winooski, VT, USA) Synergy HT plate reader. DNA was normalised to 20 ng/μL for GBS.

### 2.4. SNP Discovery and Genotyping

SNP discovery was mediated by reduced representation sequencing following the approach of Elshire et al. [18]. Briefly, DNA from each sample was digested with the restriction enzyme *ApeKI* that has a 5 bp recognition site. Digested DNA was ligated to adaptors containing a unique DNA barcode and 96 samples were then pooled to generate a single library. Each library was amplified via PCR, quantified and evaluated on a BioAnalyser prior to sequencing. Each library was sequenced on 2 lanes of an Illumina (Cambridge, UK) HiSeq 2500 to generate single-end (SE) reads of 100 bp.

Sequence data obtained with each library was concatenated and adaptor contamination was removed with Scythe [19] with a prior contamination rate set to 0.30. Sickle [20] was used to trim reads when the average quality score in a sliding window (of 20 bp) fell below a phred score of 20 and reads shorter than 40 bp were discarded. The reads were demultiplexed using Sabre [21] allowing a single mismatch, data output per sample was determined, and reads from each sample were aligned to v4.04 of the PGSC pseudomolecules [22,23] using BWA [24]. The Genome Analysis Tool Kit (GATK) [25] was used to identify putative SNPs in the population, and only biallelic SNPs with a mean mapping quality of 30 were retained for further analysis. Samples which returned less than 250,000 reads were excluded from further analysis.

For the diploid MCD population Vcftools [26] was used to retain SNPs with a minimum genotype quality of 50, a minimum depth of 20 and where genotype calls were made in at least 75% of the individuals. Vcftools was also used to remove three individuals where data was missing for over half of the remaining variants. The GATK tool VariantstoTable [25] was used to output the genotype (GT) field from the filtered vcf file. 

For the PAM population, the GATK tool SelectVariants was used to create a vcf file from the raw database of all samples with information for the five parental samples only. The aim was to narrow down to a list of variants unique to the resistant parent, RH4X-753-3. Firstly, variants were removed if they were supported by read depths of <10 in at least one parent. Secondly, variants were removed if they were not unique to the female resistant parent, RH4X-753-3, and in the simplex configuration in that parent. GATK SelectVariants was then used to filter the raw SNP database to include only progeny samples with a minimum read count of 500,000 (3 progeny removed) and an overall DP of 1500 across the remaining 75 samples i.e., SNPs with on average 20x coverage. The GT and depth (DP) data were converted to tabular form using GATK VariantsToTable, which was then filtered with the list of variants retained from the analysis of the parental samples. Among the progeny a DP of at least 20 was required to support nulliplex calls, if less they changed to missing. Duplex calls as a result of low DP were also changed to missing. 

### 2.5. Genetic Map Construction

Joinmap v4.1 [27] was used to create genetic linkage maps in both crosses. For the MCD population, individual maps were created using segregation data derived from both parents (MCD ALL) and data only from the resistant parent (MCD MALE). For the PAM population, to address the use of several different male parents in the population, polymorphic SNPs derived from the resistant (female) parent and segregating in the simplex configuration (aaab × aaaa 1:1 segregation) were used. Simplex markers in a tetraploid exhibit the same segregation ratio as uniparentally derived heterozygous markers in a diploid (abxaa, 1:1 segregation) and can be used to create genetic maps of individual homologous chromosomes in an autotetraploid (*n* = 48 intetraploid potato) utilising software designed for mapping in diploids. To create the male genetic map of the MCD population and the PAM simplex genetic map in JoinMap 4.1, genotypes observations were translated to the DH population type. This in accordance with the MapQTL manual [28] and avoids singularity errors when analysing a CP population where all markers segregate for only one of the two parents only. Joinmap v4.1 was used to sort the markers in each linkage group (LG). A logarithm of odds (LOD) of 2.0 was used to determine the LGs. Maps were constructed using regression-based parameters and Haldane’s mapping function was used to calculate the genetic distance between the markers. Linkage maps were visualised using R package LinkageMapView [29]. 

### 2.6. Analysis of QTLs

QTL analysis was conducted with MapQTL v6 [28]. The regression algorithm was used to calculate the maximum likelihood and a 1.0 mapping step size was used. The threshold of the LOD score for significance (*p* ≥ 0.05) was determined using 1000 permutations and QTLs above the chromosome-specific threshold for that trait were deemed significant. The phenotypic variance explained (PVE) of each QTL was estimated based on the population variance found within the segregating population. Interval mapping results generated using MapQTL were exported to produce QTL plots with the ggplot2 R package [30]. 

## 3. Results

### 3.1. Evaluation of Resistance in Each Mapping Population

**Figure 1:**
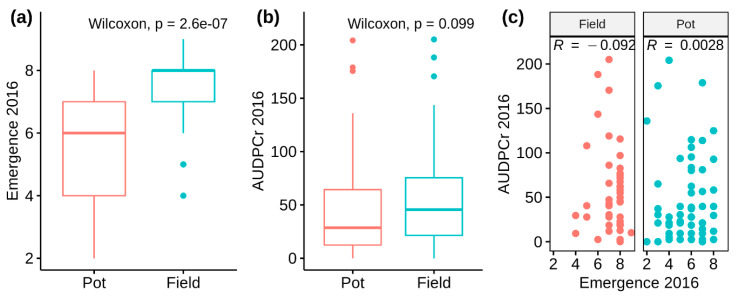
2016 *Solanum microdontum* (MCD) population field trial trait observations. (**a**) Higher emergence scores were recorded in the Field subset. (**b**) Tuber origin had no knock on effect on observed area under the disease progress curve (AUDPC) scores. (**c**) Response to late blight was not associated with emergence.

In 2016 the full *S. microdontum* (MCD) population was assessed. Two subgroups were defined by origin of the tubers: tubers harvested from the field in the previous year (Field subset) and tubers obtained from plants grown in pots (Pot subset). Emergence was scored because of an anticipated developmental difference between the subgroups. The Field subset did have higher mean (±SD) emergence scores (7.21 ± 1.17) than Pot subset (5.59 ± 1.64; Figure 1a shows distribution). However, response to late blight was not associated with either tuber origin or emergence (Figure 1b,c).

In the following years (2017 and 2019; in 2018 no blight infection observed) those in the Pot subset were assessed only. Good correlations in AUDPCr scores were observed between years (Supplementary File S2 page 2), especially between 2017 and 2019 (0.917) where scores were calculated based on observations at two reps of four tubers per genotype. Maturity was recorded in 2017 and 2019 and only a moderate correlation was observed between these scores. This is likely a reflection of the limited range of scores observed within the population and the difficulty of scoring this trait in small plots. There was no clear relationship between maturity and observed disease resistance scores. However, the development trait was negatively correlated with AUDPCr when measured in 2017 and 2019 (File S2 page 2) i.e., genotypes with well-developed foliage tended to be more resistant. 

For the *S. pampasense* (PAM) population, good correlations between AUDPC scores across years were observed in the entire population (0.453) and within each subpopulation defined by the father used in the cross to RH4X-753-3 (File S2 page 3). 

### 3.2. SNP Marker Detection and Genotyping

**Figure 2:**
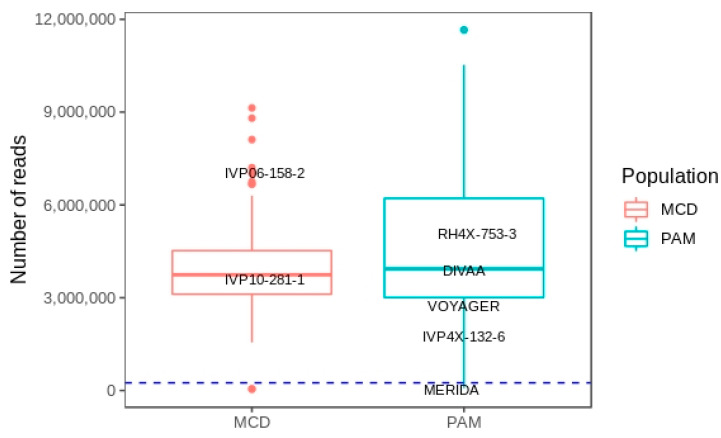
Number of reads recovered for each genotype across both the MCD and *Solanum pampasense* (PAM) derived populations. The parents in each population are highlighted. Although a relatively low number of reads were recovered for MERIDA (397,563) this sample was retained as it was the male parent of the largest subpopulation within PAM. The dashed line represents a cut off point of 250,000 reads. Two samples from the MCD population and three from the PAM population did not meet this threshold and were removed from further analysis.

A total of 462,251,665 FastQ records were generated from Illumina sequencing of the MCD library for 96 individuals of the mapping population. Sickle discarded 5,798,267 reads which were shorter than 40 bp after adaptor and quality trimming. Sabre allowed demultiplexing of the remaining reads to each of the 96 individuals. Two samples were excluded at this stage due a low number of reads (Figure 2). The 94 remaining samples (2 parents and 92 progeny) had a mean read count of 4,048,355 reads with the female susceptible and male resistant parents having 6,674,645 and 3,227,362 reads, respectively (Figure 2). 

After demultiplexing reads to individuals in the PAM population three genotypes were eliminated for low read counts (Figure 2). Of the remaining 73 samples (5 parents and 68 progeny) a mean read count of 4,860,202 was calculated. The female resistant parent, RH4X-753-3, had 4,694,994 reads while the number of reads of the male parents were as follows: DIVAA 3,494,289; VOYAGER 3,112,894; IVP4x-132-6 2,112,948; MERIDA 397,563. Although the read count for the MERIDA sample was low we did not want to exclude it as it gave rise to the largest subpopulation within PAM.

A total of 799,826 SNPs were discovered in the MCD population. After filtering for genotype quality, read depth and representation among the individuals, this number was reduced to 26,049 biallelic SNPs. Three of the progeny were removed from analysis at this point where data was missing for over half of the retained SNPs. After filtering out those SNPs in which a parent had missing data or if incompatible genotypes were present among the progeny (which would indicate an error in a parental genotype call) an SNP set of 12,133 was available. Variants with identical genotype calls across each of the remaining progeny were removed. At this stage a further five individuals where over 25% of the remaining 9570 variants had missing data were removed. A further 2488 loci with greater than 10% missing data among the remaining 84 progeny were removed. After filtering to remove severely distorted markers, 1390 IVP10-281-1/resistant parent derived markers, 1010 IVP06-158-2/susceptible parent markers and 748 biparentally inherited markers remained for mapping. Each marker was named according to the chromosome and position on the reference genome.

A total of 1,345,378 SNPs were discovered in the PAM population. The sequence data generated generally does not provide the consistent read depth (60–80x [31]) required to reliably determine dosage which would facilitate utilisation of multiple dosage states in mapping. As a result of this, and the added complexity of the PAM population (one female and four male tetraploid parents (MERIDA, IVP4x-132-6, VOYAGER, DIVAA)), we identified only simplex (single dose) variants from the resistant parent and used these for generation of a maternal genetic map covering the individual homologous chromosomes of this tetraploid genotype. We first examined the genotype calls for each of 92,263 detected variants for the parents only. Variants were removed if they were supported by read depths of <10 in at least one parent (down to 8275). This was set deliberately low as read depths at each locus for the MERIDA parental sample were consistently low due to the total number of reads recovered (Figure 2). Variants were only retained if they were genotyped as simplex in RH4X-753-3 and nulliplex in each male parent (1986 markers) or triplex in RH4X-753-3 and quadriplex in each male parent (10 markers). Variants with identical genotype calls across each of the 68 genotypes in the population were removed leaving a set of 1419 variants for mapping. Using this approach single dosage markers of the resistant parent could be used for linkage mapping in a multi-parent tetraploid population directly in JoinMap 4.1. 

For the PAM population, the GATK tool SelectVariants was used to create a vcf file for SNP variants from the raw database with information for the five parental samples only. The aim was to narrow down to a list of variants unique to the resistant parent, RH4X-753-3. Subsequently, variants were removed if they were common to all parents, or unique to one or more of the male parents. As a result that read depths at each locus were low for MERIDA, the remaining variants were filtered for a minimum read depth of 10 in each parent.

### 3.3. Linkage Map Construction

High-density biparental linkage maps for the MCD diploid population were constructed both with 1431 SNP markers derived from both parents (MCD ALL, File S1) and 752 SNP markers unique to the resistant parent (MCD MALE, File S1). Linkage groups covering all 12 chromosomes of potato were defined and identified (Table 1) on the basis of SNP positions on the pseudomolecule assembly v4.04 of the potato genome sequence [22,23]. 

For the PAM population 967 simplex SNP markers from the resistant parent grouped into 46 linkage groups, representing individual homologous copies of the 12 parental chromosomes (Table 2, File S1). These were assigned letters based on decreasing numbers of markers i.e., chr01A represents the linkage group corresponding to chromosome 01 with the most number of markers followed by chr01B, chr01C and chr01D. Typically two well populated linkage groups per chromosome were constructed with these resistant parent specific SNPs which are likely to represent those with introgression segments from the orginal diploid *S. pampasense* donor. These are probably representative of low frequency or novel variants relative to the cultivated European *S. tuberosum* gene pool in the population since SNPs common to the multiple male parents were removed during the filtering process. In some case homologues are fragmented resulting in linkage groups labelled “E”. Six homologues are unrepresented (two for chr05 and chr11, one each for chr04 and chr06). In general, markers occurred in concordance with their expected order relative to their assumed physical position on the physical map (File S1), meaning map quality is reasonable. However, it is clear that complete coverage of all homologues was not achieved, and this will impact the ability to detect QTLs in regions lacking coverage.

The sequences on chr00 of the potato reference genome represent unanchored sequences. Often SNPs identified within these contigs were mapped in the three genetic maps reported in this study. This allows us to putatively anchor these contigs to individual chromosomes and possible physical locations based on co-segregation with SNP markers on anchored sequences (File S1).

### 3.4. QTLs for Late Blight Resistance in MCD Diploid Population

**Figure 3:**
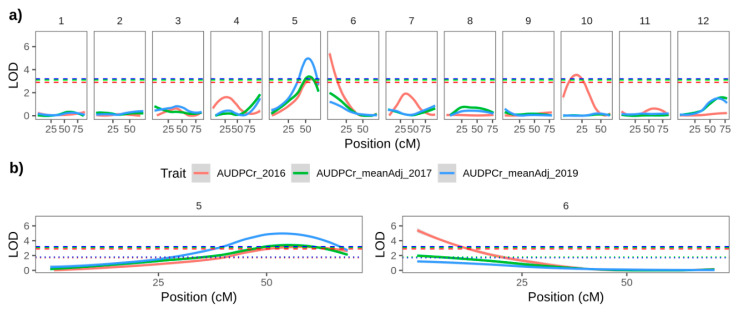
Interval mapping results using the MCD MALE map and each year’s AUDPC data for (**a**) all linkage groups and (**b**) linkage groups corresponding to chromosomes 5 and 6. Red, green and blue solid lines represent logarithm of odds (LOD) plots for 2016, 2017 and 2019, respectively. Similarly coloured dotted lines represent the chromosome-wide permutation threshold for that year, and dashed lies represent the more stringent genome-wide permutation threshold for the same year.

We performed interval mapping using both the MCD ALL and MCD MALE maps. Results were similar for both, (a slightly greater number of QTLs was detected in the MCD MALE map) and for simplicity only the loci detected using the MCD MALE map are presented below. Potential QTL were declared if the associated LOD score exceeded the chromosome-specific permutation threshold in any year, and we further distinguish between consistent effects, discovered in the same position over multiple seasons and less robust effects detected in only one season. A consistently significant QTL for late blight resistance was detected at the beginning of the linkage group corresponding to chromosome 06 (Figure 3b, Table 3). This locus exceeded the chromosome specific LOD thresholds in 2017 and the more stringent genome-wide threshold in 2016, explaining. These loci explained 30.8% and 20.7% of the phenotypic variation in 2016 and 2017, respectively. (Figure 3a,b, Table 3). 

In addition, a consistent effect was detected using each year’s AUDPC data on linkage groups corresponding to chromosome 5 (Figure 3b; File S2 page 5; Table 3). This locus exceeded the genome-wide threshold in all three years (Figure 3b) explaining 21.1%, 33.9% and 47.3% of the variation in 2016, 2017 and 2019, respectively (Table 3). Each year the peaks of this QTL are at 61–68 cM on SNP markers with a physical location at approximately 50 Mb. This is over 40 Mb from the *CDF1* locus at 4.5 Mb [32], the transcription factor gene associated with early maturity and initiation of tuberization [33]. The correlation between field resistance to late blight and late maturity is well known [34]. However, the failure to detect QTLs on chromosome 05 with the maturity data collected (File S2 pages 4 and 6) and the physical distance between the detected late blight resistance QTL and *CDF1* suggest that the effect is not due to maturity type.

A further locus explaining variation in late blight resistance was detected on the linkage group corresponding to chromosome 10 with the full population in 2016 only. This locus corresponded to a consistent effect detected in 2017 and 2019 that was also responsible for variation in foliar development (Table 3 and File S2 page 4–6). Well developed plants tended to be more resistant to blight infection. Finally a weak, but inconsistent effect was detected on a linkage group corresponding to chromosome 8 in 2019. 

### 3.5. QTLs for Late Blight Resistance in PAM Multi Parent Tetraploid Population

**Figure 4:**
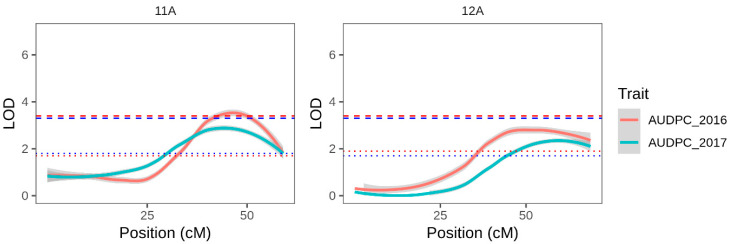
Interval mapping results using the PAM map and each years AUDPC data for linkage groups corresponding to chromosomes 11 and 12, where QTLs exceeding the chromosome specific LOD permutation threshold were found in 2016 and 2017. Red and blue solid lines represent LOD plots for 2016 and 2017 respectively. Similarly coloured dotted lines represent the chromosome-wide permutation threshold for that year, and dashed lies represent the more stringent genome-wide permutation threshold for the same year. Plots for the additional 43 linkage groups can be seen in File S2 page 7.

Interval mapping was carried out with the 46 linkage groups constructed with single dose markers unique to the female parent. Consistent effects (Table 4, Figure 4) were detected in both years at the end of linkage groups corresponding to chromosomes 11 and 12. The chr11 and chr12 QTL explained 22.6% and 20.8% of the variation in 2016, respectively. Although the correlation in AUDPC scores between years was moderate (0.453; File S2 page 3), the same two QTLs were detected in 2017 explaining 19 and 16.9% of the variation.

A QTL for late blight resistance was detected at the beginning of a linkage group corresponding to chromosome 4 with the 2016 phenotypic data only (Table 3). The peak LOD was at 13.4 cM on a SNP marker with a physical location at 5.1 Mb. This corresponds to location where the *R2* gene from *S. demissum* has been mapped [35]. However, this locus showed no association with the resistance scores in 2017.

### 3.6. Potential to Use Detected Variants in MAS for Late Blight Resistance

The advantage of QTL analysis with sequence-based markers is that variants can be readily converted to SNP-based molecular marker assays for use in MAS. Both the *S. microdontum* and *S. pampasense* populations exhibited pairs of individual QTLs detectable over multiple years, on Chromosomes 5 and 6 in the case of MCD, and Chromosomes 11 and 12 in the case of PAM. The partial nature of the resistance conferred by the loci means that a MAS-based stacking approach would be the most appropriate means of deployment, especially if the pairs of loci exhibited a consistently additive effect within their respective populations over the multiple test years. To test the potential effect of selecting for these consistently effective pairs of loci within the existing crosses we constructed simple genetic models using individual coupling-phase SNP markers with the largest effect on the trait value as indicated by a Kruskal–Wallis analysis. 

For MCD, in 2016 the heterozygous genotype of chr06_7,264,151 at 18.0 cM on the MCD ALL chr06 map (5.4 cM on MCD MALE chr06 map) was associated (K * 19.5, *p* < 0.0001) with lower mean AUDPC scores (mean aa = 85.5; mean ab = 39.4). Likewise, in 2016 the heterozygous genotype of chr05_47,763,981 at 56.0 cM on the MCD ALL chr05 map (53.1 cM on MCD MALE chr05 map) was associated (K * 14.8, *p* < 0.0005) with lower mean AUDPC scores (mean aa = 74.2; mean ab = 32.2). 

Within the PAM population in 2016, the heterozygous genotype of chr11_44,857,837 at 47.2 cM on the PAM chr11A map was associated (K * 15.7, *p* < 0.0001) with lower mean AUDPC scores (mean a = 1154.1; mean b = 622.7). Similarly, the heterozygous genotype of chr12_57,706,386 at 61.7 cM on the PAM chr12A map was associated (K * 14.9, *p* < 0.0005) with lower mean AUDPC scores (mean a = 1150; mean b = 608.4). 

**Figure 5:**
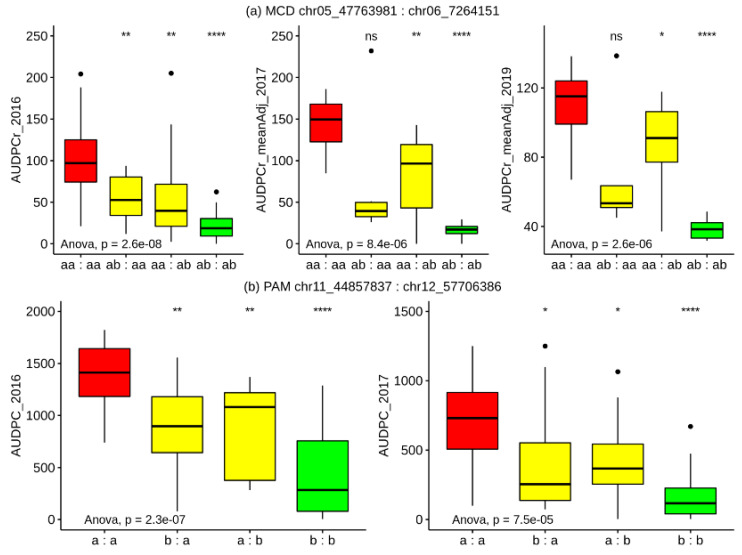
Effects of combinations of alleles derived from *S. microdontum* (**a; top**) and *S. pampasense* (**b; bottom**) on late blight disease resistance over multiple growing seasons. Whisker plots show the mean AUDPC and quartile ranges for each allele combination (indicated in the title at the top of each panel labelled (**a**,**b**)). On the *x*-axis, “a” indicates the susceptible allele and “b” indicates the resistant allele; the combination is represented in the order of the panel title. Two significant QTLs were detected in each population. Genotypes with alleles from the resistant source at each locus (green) had the lowest mean AUDPC i.e., they were the most resistant group. The *p*-values for one-way ANOVA test comparing each group within each trial are displayed on the bottom left of each plot. Furthermore, the *p*-values obtained from multiple pairwise tests against the reference group (red, neither resistance allele present) are indicated above each plot: ns *p* > 0.05; * *p* ≤ 0.05, ** *p* ≤ 0.01, *** *p* ≤ 0.001, **** *p* ≤ 0.0001.

Simple genetic models showing the effect of possessing all four possible combinations (neither resistance allele, exclusively one or the other resistance allele, or both alleles) in each of the populations are illustrated in Figure 5. For both populations, the distribution of observed AUDPC in genotypes with both resistance alleles is consistently lowest across all test years, indicating that the pairs of loci within each population are exhibiting an additive effect, and that a MAS-based stacking approach targeting these loci is valid.

## 4. Discussion

This study was carried out in the context of a larger initiative to introgress late blight resistance from wild species sources into the elite cultivated potato genepool. Our goal was to increase the potential efficiency of this process by identifying SNP markers that were diagnostic for loci underlying the quantitative resistance in the two sources examined (MCD and PAM), and to partially validate these polymorphisms for their utility in MAS-driven pyramiding approaches. We were somewhat constrained by the available seed population sizes available and there were some sub-optimal features to the experimental design because of this and other operational constraints. For the MCD source, in the first year (2016), in which 106 individuals were used, plots were unreplicated due to low tuber numbers. For the two subsequent test years (2017 and 2019) it was only possible to use 58 and 51 genotypes, respectively, although these were subject to replication. For the PAM source, a composite population (*n* = 71) consisting of smaller populations (*n* = 19, 20, 21 and 11) from crosses between the resistant (maternal) parent RH4X-753-3 and the susceptible paternal parents MERIDA, IVP4x-132-6, VOYAGER, DIVAA was used, and trials were unreplicated in both 2016 and 2017.

To mitigate against these features, we adopted an approach whereby late blight resistance QTLs were only declared when the chromosomal or genome-wide permutation threshold was exceeded in the same position in two or more years, supporting the existence of robust and consistently expressed effects. Using this approach, two late blight resistance QTLs were consistently identified in the MCD parent over multiple years, on chromosomes 5 and 6 (Figure 3 and Table 3). A further two QTLs were consistently identified in the PAM parent on chromosomes 11 and 12 over 2 years (the PAM population was not phenotyped in 2019).

The sequence-based nature of our genotyping allows us to assign genome co-ordinates to SNP markers on the pseudochromosome molecule assembly of DM. In many cases, the peak of the QTL effect coincides with the known position of NB-LRR R-gene clusters in the reference sequence [36], and we hypothesise that the resistance effect in these cases is mediated by partially effective R-genes.

Resistance QTL from a *S. microdontum* source were detected on chr05 in a previous study, although inconsistently [10]. This may be analogous to the chr05 QTL also detected in this study. A number of R genes with homology to *Rpi-blb2* also exist between 41.7–49.6 Mb of chr05 [36,37]. Resistance QTL from this species have been described previously from chr04 and chr10 [10]. *Rpi-mcd1* was mapped to chr04 [12]. The consistent QTL detected on chromosomes 5 & 6 using a mapping population derived from a new *S. microdontum* source (MCD 360-8) may be a novel basis of resistance to late blight. The region in which the chr06 QTL was detected represents a small number of NB-LRR gene clusters [36] in which genes such as *Rpi-blb2* [38] and *Rpi-ver1* [39] have also been mapped. QTLs for resistance to *P. infestans* and *Erwinia carotovora* (subsequently renamed *Pectobacterium carotovorum)* have been described in this region [40,41,42]. Similar to the *S. microdontum* source used by Bisognin et al. [11], the late blight resistance from the MCD 360-8 source was not correlated with maturity and should be a useful source of resistance to consider for breeding. 

Consistent QTL were also detected on chr11 and chr12 in the PAM parent. The markers at the peak of the chr11 QTL have proximity to a number of *I2* homologues in a divergent resistance gene cluster present in both potato and tomato in which several resistance traits have been mapped [43]. In potato, these include the late blight resistance genes *R3a* [44], *R3b* [45], *R6/R7* [46], *R10/R11* [47] from *Solanum demissum*, *Rpi-sto2* [48] from *Solanum stoloniferum* and *Rpi-smira1* from blight resistant cultivar SARPO MIRA [49]. The peak of the chr12 QTL have proximity to a number of *R1*-type R genes and other TOLL/interleukin-1 receptor NB-LRR genes [37]. QTL for late blight resistance have been described at the distal end of this chromosome previously [41,50,51]. In addition, resistance genes for *G. pallida* (*Gpa2* [52]) and potato virus X (*Rx* [53]) have been found at this “hot spot” for R genes [54]. SNP markers identified in this study may therefore have proximity to novel resistance genes from the *S. pampasense* source. 

In addition to the QTLs detected in the populations over multiple years, some additional loci were detected in only one year. In the MCD parent, a QTL for late blight resistance was detected on chr10 in 2016 only. Although it fell below our requirements to declare a consistently effective QTL it is of some interest because it overlapped with a QTL for foliar development that was discovered in both 2017 and 2019 (Table 2, File S2 page 4). Although there are some NB-LRR genes in this region of chr10 [36], it is more likely that the apparent resistance to late blight associated with the locus in the population is related to foliar development, with bushier plants tending to have lower AUDPC scores. Alternatively, this could be the same QTL detected previously in a different source of *S. microdontum* [10], or, since it was detected only in one year, it may be a spurious observation.

In the PAM population, a QTL exceeding the permutation threshold was found in 2016 only. Again, despite not meeting the criteria in our study for the declaration of a consistent effect, it is noteworthy that the markers at the peak of this QTL are in a similar position to where *R2* from *S. demissum* has been mapped [35]. The PAM parent (RH4X-753-3) is known to possess *R2*, donated by one of the tetraploid cultivars in its pedigree. Numerous other homologous late blight resistance genes including *Rpi-blb3*, *R2-like* and *Rpi-abpt* [55,56] have also been mapped at this position. Thus, it is possible that the effect observed in this year is due to *R2*. However, this locus did not explain any of the variation in resistance the following year. This may have been due to a number of reasons including the decrease in the mapping population size in subsequent years limiting our ability to detect the effect, a change in the effector profile of the prevalent late blight population in the subsequent years or the possibility that this is simply a spurious observation.

It is increasingly accepted that best practice for the deployment of R-gene mediated disease resistance in varietal development involves avoiding the release of R-genes on an individual basis, instead favouring stacking multiple R-genes in variety candidates. On a molecular basis, the presence of multiple R-genes interacting with different effectors of the same pathogen potentially provides more durable resistance by making it more difficult for pathogen populations to adapt. Interestingly, adopting this strategy means that partially effective R-genes may also have a role to play, stacked either with fully effective R-genes or with complimentary partially effective loci with which they demonstrate an additive effect, as in this study. This suggests that, despite the availability of a fairly large number of fully and partially effective loci for resistance to late blight already, continuing to characterize potential novel loci and introgress them into an elite background is a valuable activity. The availability of a very large pool of resistance loci with different specificities, combined with the potential for MAS-driven stacking, and other rapid breeding approaches, could result in the release of cultivars with a diverse array of different R-gene stacks over relatively short periods of time, potentially increasing the useful lifespan of the resistance loci in question through a phenomenon of cross-protection.

In general, it is difficult to effectively combine multiple resistance genes in the manner described above in the absence of molecular markers. Although we have not independently validated the effectiveness of a MAS driven strategy for the loci identified in this study by transferring the loci into a different background, the simple genetic models built on the most suitable SNPs identified during QTL mapping clearly demonstrate its potential. Future work will involve development of cost effective and high throughput molecular marker assays allowing MAS-based stacking to be adopted for these loci. The PAM population is already at the tetraploid level, and arguably closer to useful deployment in breeding, whilst the MCD population needs to cross the ploidy barrier; in both cases, MAS will allow the co-retention of effective loci, and these can even be further stacked with other effective loci using MAS. The latter might be important in the case of the PAM population, since the overall levels of resistance, even employing both loci, seemed lower in that population, although it is worth noting that the two populations were tested in different environments, so such direct comparisons are not necessarily valid. Finally, we have not specifically addressed the broadness of effect of these partially effective loci, however there is evidence that partially effective loci from similar sources exhibit broad spectrum resistance; e.g., *Rpi-mcd1* is thought to be a weak but broad-spectrum resistance gene [12]. Further work is required to confirm the broadness of effect of the QTLs identified in this study. 

## Figures and Tables

**Table 1:** Summary of mapping results with the MCD population. Summaries are given for both the MCD ALL and MCD MALE maps, described in the text. For each chromosome the number of markers mapped on each linkage group is shown on the left. The genetic distance of each map is shown on the right. The MCD ALL map was created all available single nucleotide polymorphism (SNP) markers. The MCD MALE genetic map was constructed with markers originating from the male parent only.

	Number of Markers		Genetic Size (cM)	
Chromosome	MCD ALL	MCD MALE	MCD ALL	MCD MALE
**1**	140	88	88.8	88.7
**2**	142	69	104.2	70.3
**3**	91	62	89.7	85.6
**4**	148	65	94.0	96.2
**5**	118	74	91.9	68.7
**6**	119	44	83.1	70.9
**7**	114	71	63.3	94.5
**8**	140	62	101.7	76.8
**9**	148	52	100.3	79.2
**10**	53	48	56.8	62.5
**11**	110	66	81.1	85.9
**12**	108	51	69.5	78.2
**Total**	1431	752	1024.4	957.5

**Table 2:** Summary of mapping results with the PAM population. Simplex markers unique to the female resistant parent, RH4X-753-3, were used for mapping. For each chromosome the number of markers mapped on each linkage group is shown on the left. The genetic distance of each map is shown on the right. A–E represent homologous linkage groups as described in the text.

	Number of Markers					Genetic Size (cM)				
Chromosome	A	B	C	D	E	A	B	C	D	E
**1**	52	13	8	6	-	36.7	58.2	64.3	22.5	-
**2**	49	17	8	6	4	86.3	59.2	1.6	51.5	28.4
**3**	42	15	11	7	5	66.3	31.9	38.0	22.5	33.7
**4**	36	19	17	-	-	59.2	50.8	57.2	-	-
**5**	31	18	-	-	-	24.3	104.6	-	-	-
**6**	38	37	29	-	-	41.8	117.4	96.3	-	-
**7**	51	41	23	6	5	69.5	141.8	106.4	14.2	8.8
**8**	49	22	8	3	-	35.0	55.0	30.1	30.2	-
**9**	60	21	11	7	-	69.2	78.4	81.5	29.8	-
**10**	34	33	4	4	-	113.6	39.8	18.0	6.8	-
**11**	37	16	-	-	-	59.0	79.2	-	-	-
**12**	40	8	8	5	3	68.8	48.3	49.3	43.5	3.9
**Total**	967					2432.6				

**Table 3:** Quantitative trait loci (QTL) analysis of late blight resistance and associated traits in the MCD MALE map. LG, linkage group; Position, position on LG; LOD, logarithm of odds; PVE, phenotypic variance explained by QTL. Potential QTLs were declared if LOD scores exceeded the appropriate calculated LOD thresholds for each LG and each trait (1000 permutations, *p*-value 0.05). Consistent QTLs appearing in similar positions in multiple seasons are indicated in normal type, effects detected only in one season are indicated in italics. SNP marker names refer to the chromosome and physical location on the reference genome.

Trait	LG	Peak LOD Position (cM)	LOD	1.5 LOD Support Intervals (cM)	Marker Interval	PVE (%)
AUDPCr 2016	chr05	53.7	3.81	44.8–68.7	chr05_9,405,130-chr05_51,443,752	21.1
	chr06	1.2	6.70	0–1.3	chr06_250,131-chr06_2,026,583	30.8
	*chr10*	*20*	*4.14*	*6.5–26.3*	*chr10_1,742,105-chr10_55,584,190*	*20.3*
AUDPCr meanAdj 2017	chr05	57.9	3.77	44.8–66.5	chr05_9,405,130-chr05_50,632,205	33.9
	chr06	1.2	2.11	0–29.3	chr06_250,131-chr06_42,886,351	20.7
AUDPCr meanAdj 2019	chr05	54.7	5.18	44.7–61.8	chr05_9,405,130-chr05_50,078,823	47.5
Development 2017	chr10	20.9	4.85	17.9–27.7	chr10_48,765,420-chr10_51,266,814	41.2
Development 2019	chr10	22.9	2.49	0–57.0	chr10_314,470-chr10_58,392,456	26.6
Maturity 2019	*chr08*	*36.2*	*1.95*	*3.0–76.8*	*chr08_3,736,588-chr08_56,535,009*	*21.5*

**Table 4:** Quantitative trait loci analysis of late blight resistance and associated traits in the PAM population. LG, linkage group; Position, position on LG; LOD, logarithm of odds; PVE, phenotypic variance explained by QTL. QTLs were declared if LOD scores exceeded the appropriate calculated LOD thresholds for each LG and each trait (1000 permutations, *p*-value 0.05). QTLs appearing in similar positions in multiple seasons are indicated in normal type, effects detected only in one season are indicated in italics. SNP marker names refer to the chromosome and physical location on the reference genome.

Trait	LG	Peak LOD Position (cM)	LOD	1.5 LOD Support Intervals (cM)	Marker Interval	PVE (%)
AUDPC 2016	*chr04A*	*13.4*	*3.67*	*10.3–21.0*	*chr04_389,462-chr04_60,508,799*	*22.0*
	chr11A	44.6	3.78	35.1–57.2	chr11_41,141,856-chr11_44,857,837	22.6
	chr12A	61.7	3.44	43.4–68.8	chr12_28,908,129-chr12_59,023,423	20.8
AUDPC 2017	chr11A	39.6	3.11	30.9–59.0	chr11_38,030,822-chr11_42,357,437	19
	chr12A	61.7	2.74	42.7–68.8	chr12_28,908,129-chr12_59,676,913	16.9

## Supplementary Materials

The following are available online at https://www.mdpi.com/2073-4425/11/7/732/s1: File S1: Genetic maps created using SNP markers, File S2: Trait correlations and additional interval mapping results.
