# Supplementary material for: Detection of Novel QTLs for Late Blight Resistance Derived from the Wild Potato Species Solanum microdontum and Solanum pampasense
Source: Genes (Basel). 2020 Jun 30;11(7):732. doi: 10.3390/genes11070732 (PMC7396981; doi:10.3390/genes11070732)
Supplement: Supplementary file 1 [file genes-11-00732-s001.zip › Supplementary (revised_)/S1.pdf]

Detection of novel QTLs for late blight resistance derived the wild potato species *Solanum microdontum* and *Solanum pampasense*

Genes

Fergus Meade, Ronald Hutten, Silke Wagner, Vanessa Prigge, Emmet Dalton, Hanne Grethe Kirk, Denis Griffin, Dan Milbourne

Correspondence: dan.milbourne@teagasc.ie

Genetic maps created using SNP markers identified using Genotyping-by-Sequencing of each population

Page 1-2: Genetic map created using 1431 SNP markers of all types (segregating in the female parent <lmxll>; segregating in both parents <hkxhk>; segregating in the male parent <nnxnp>) of the MCD diploid population.

Page 3-4: Genetic map created using 752 SNP markers unique to the male parent of the MCD diploid population.

Page 5-16: Genetic map created using 967 SNP markers unique to the female parent of the PAM tetraploid population.

Page 17: Assignment of chr00 contigs to chromosomes based on MCD and PAM genetic maps.

MCD genetic map (All marker types, chr01−chr06)

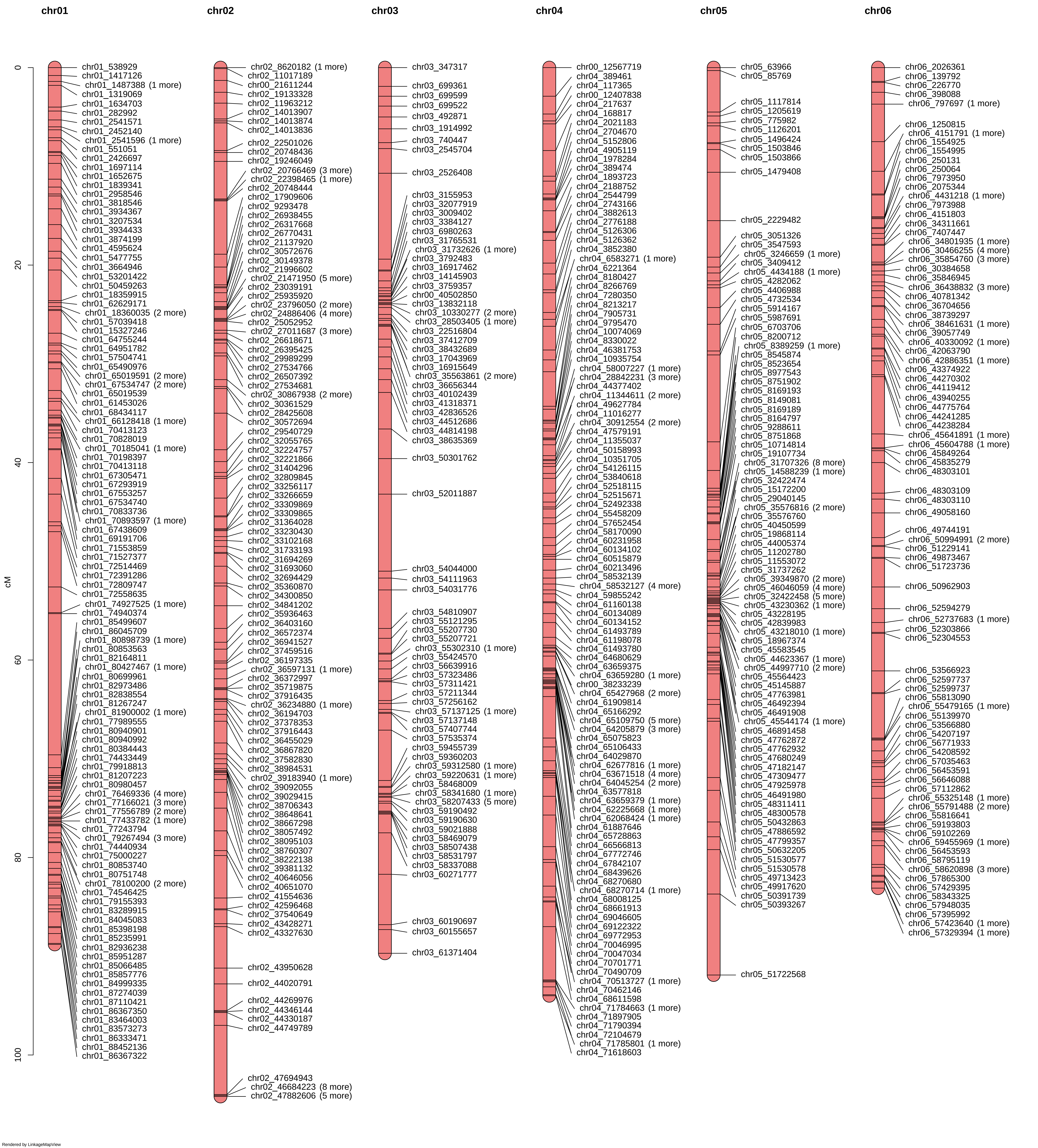

MCD genetic map (All marker types, chr07−chr12)

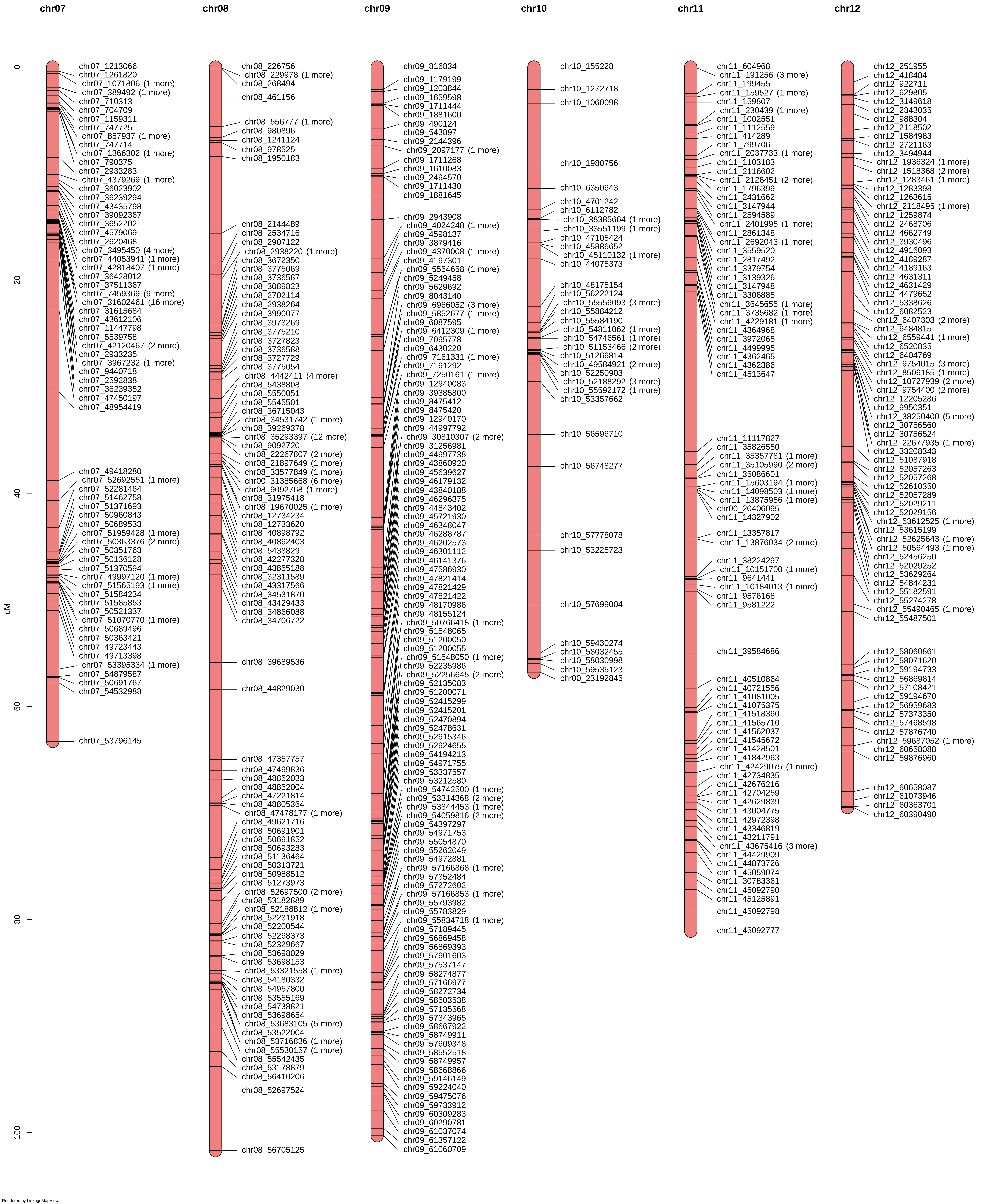

MCD MALE genetic map (markers unique to IVP10−281−1, chr01−chr06)

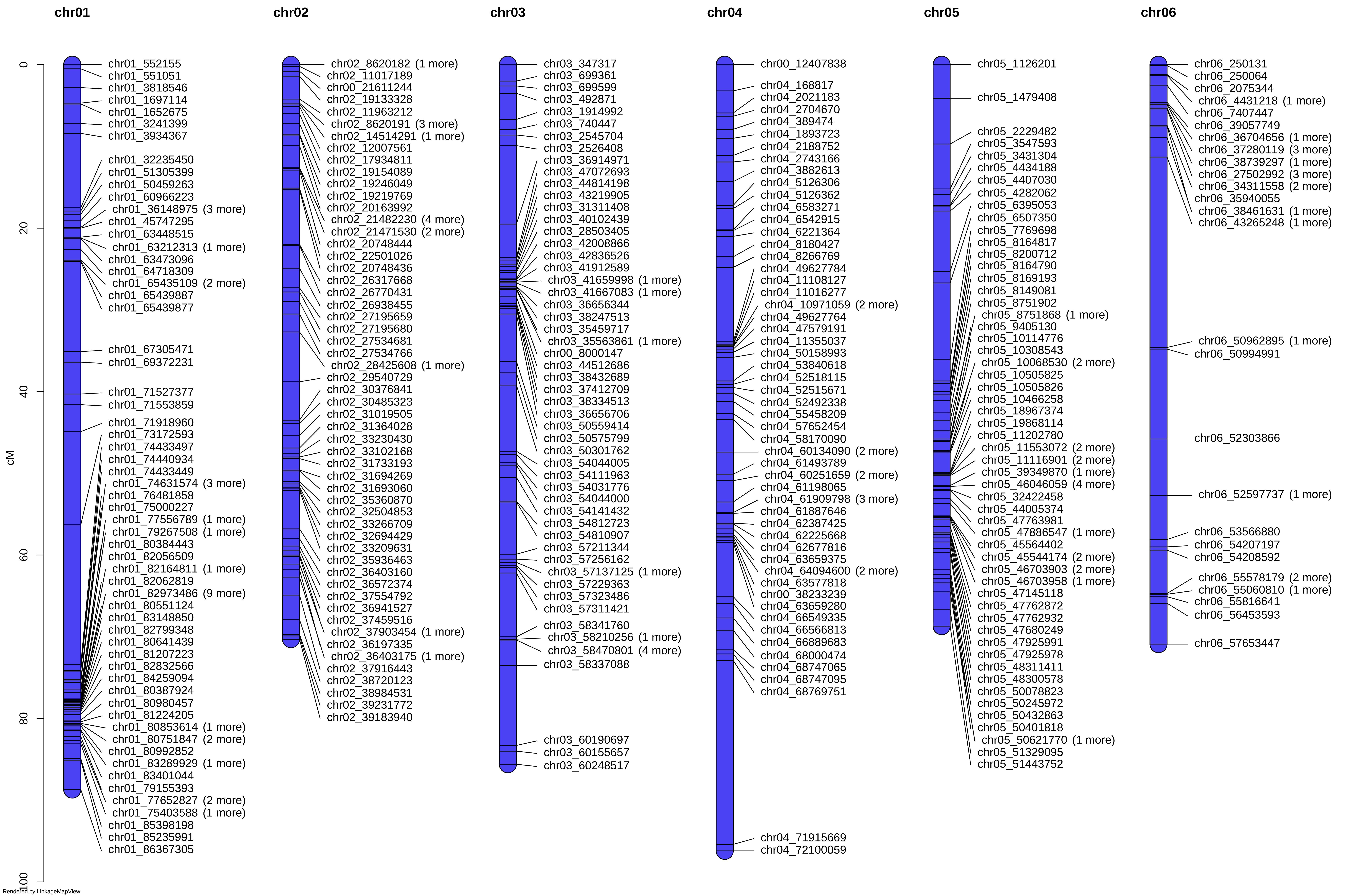

MCD MALE genetic map (markers unique to IVP10−281−1, chr07−chr12)

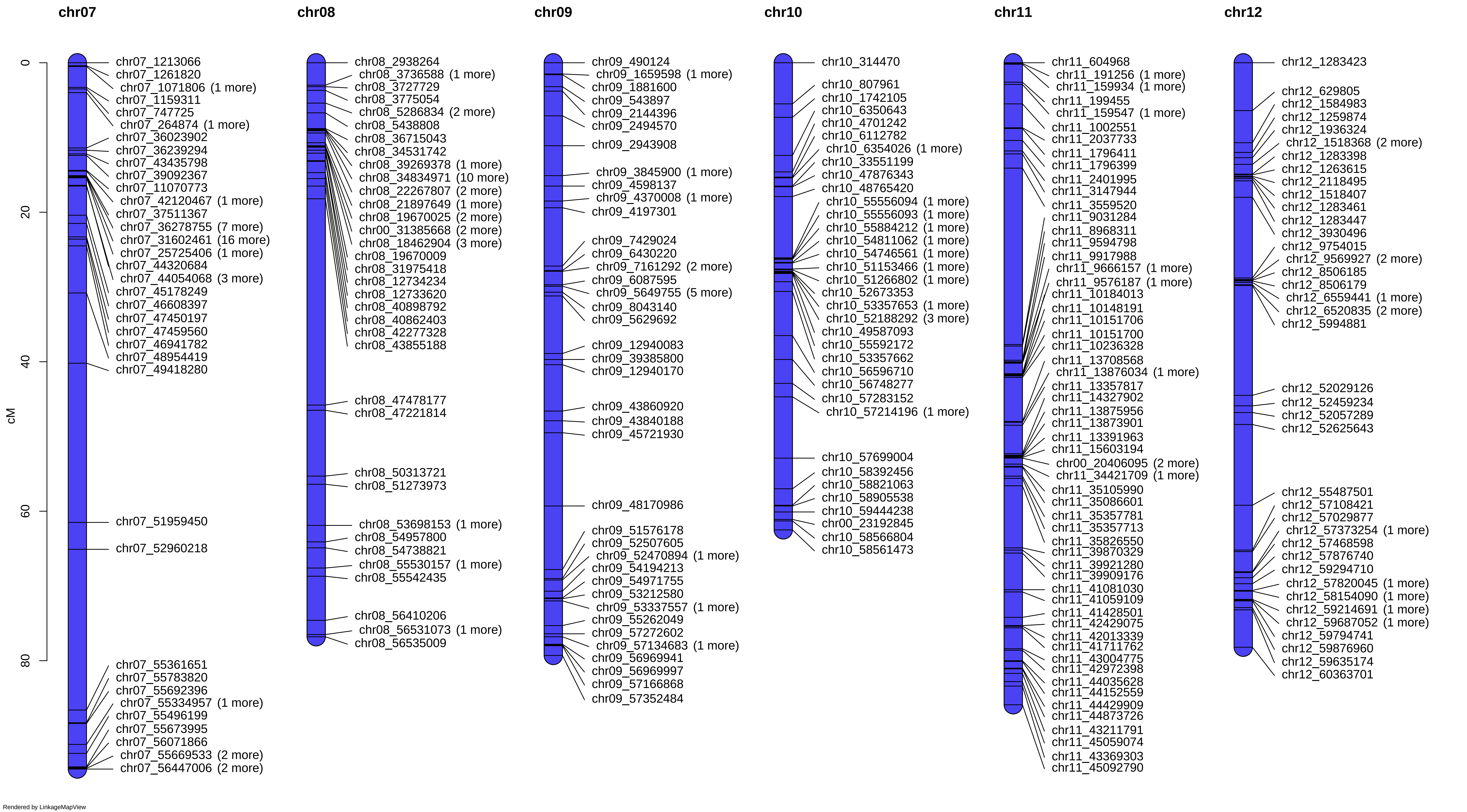

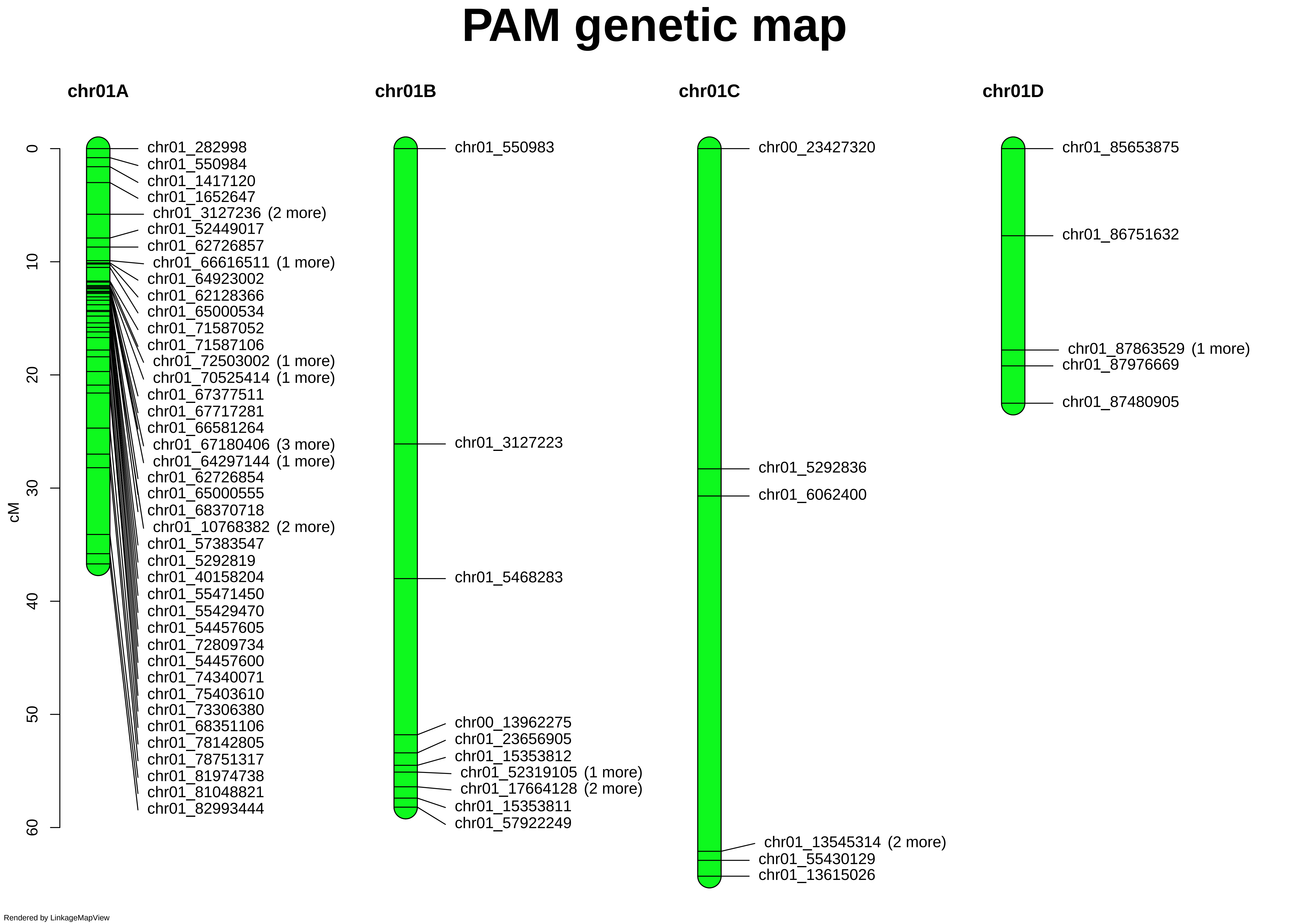
PAM genetic map
chr01A
chr01B
chr01C
chr01D
cM
0
10
20
30
40
50
60
chr01_282998
chr01_550984
chr01_1417120
chr01_1652647
chr01_3127236 (2 more)
chr01_52449017
chr01_62726857
chr01_66616511 (1 more)
chr01_64923002
chr01_62128366
chr01_65000534
chr01_71587052
chr01_71587106
chr01_72503002 (1 more)
chr01_70525414 (1 more)
chr01_67377511
chr01_67717281
chr01_66581264
chr01_67180406 (3 more)
chr01_64297144 (1 more)
chr01_62726854
chr01_65000555
chr01_68370718
chr01_10768382 (2 more)
chr01_57383547
chr01_5292819
chr01_40158204
chr01_55471450
chr01_55429470
chr01_54457605
chr01_72809734
chr01_54457600
chr01_74340071
chr01_75403610
chr01_73306380
chr01_68351106
chr01_78142805
chr01_78751317
chr01_81974738
chr01_81048821
chr01_82993444
chr01_550983
chr01_3127223
chr01_5468283
chr00_13962275
chr01_23656905
chr01_15353812
chr01_52319105 (1 more)
chr01_17664128 (2 more)
chr01_15353811
chr01_57922249
chr00_23427320
chr01_5292836
chr01_6062400
chr01_13545314 (2 more)
chr01_55430129
chr01_13615026
chr01_85653875
chr01_86751632
chr01_87863529 (1 more)
chr01_87976669
chr01_87480905
Rendered by LinkageMapView

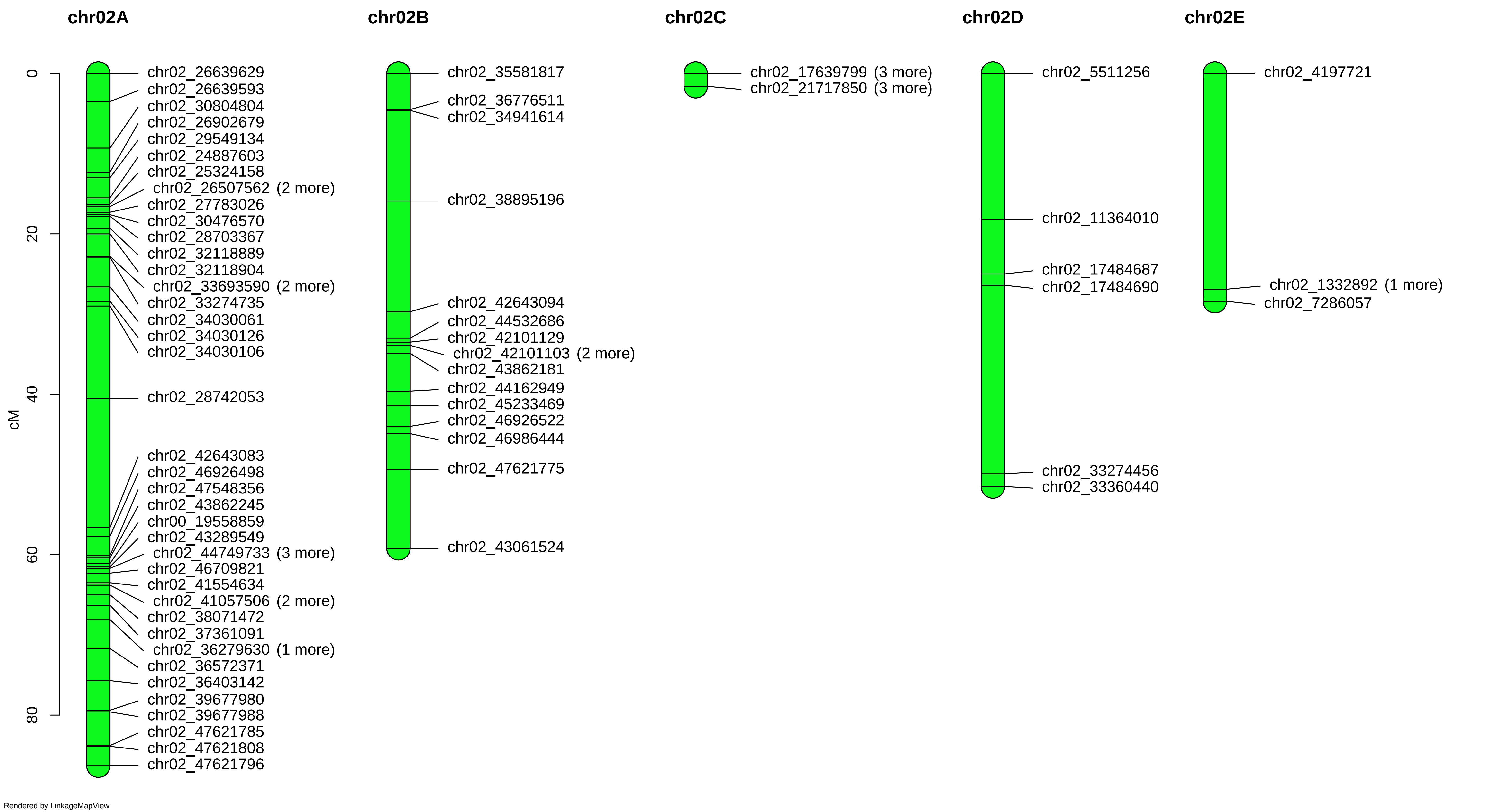
chr02A
chr02B
chr02C
chr02D
chr02E
cM
0
20
40
60
80
chr02_26639629
chr02_26639593
chr02_30804804
chr02_26902679
chr02_29549134
chr02_24887603
chr02_25324158
chr02_26507562 (2 more)
chr02_27783026
chr02_30476570
chr02_28703367
chr02_32118889
chr02_32118904
chr02_33693590 (2 more)
chr02_33274735
chr02_34030061
chr02_34030126
chr02_34030106
chr02_28742053
chr02_42643083
chr02_46926498
chr02_47548356
chr02_43862245
chr00_19558859
chr02_43289549
chr02_44749733 (3 more)
chr02_46709821
chr02_41554634
chr02_41057506 (2 more)
chr02_38071472
chr02_37361091
chr02_36279630 (1 more)
chr02_36572371
chr02_36403142
chr02_39677980
chr02_39677988
chr02_47621785
chr02_47621808
chr02_47621796
chr02_35581817
chr02_36776511
chr02_34941614
chr02_38895196
chr02_42643094
chr02_44532686
chr02_42101129
chr02_42101103 (2 more)
chr02_43862181
chr02_44162949
chr02_45233469
chr02_46926522
chr02_46986444
chr02_47621775
chr02_43061524
chr02_17639799 (3 more)
chr02_21717850 (3 more)
chr02_5511256
chr02_11364010
chr02_17484687
chr02_17484690
chr02_33274456
chr02_33360440
chr02_4197721
chr02_1332892 (1 more)
chr02_7286057
Rendered by LinkageMapView

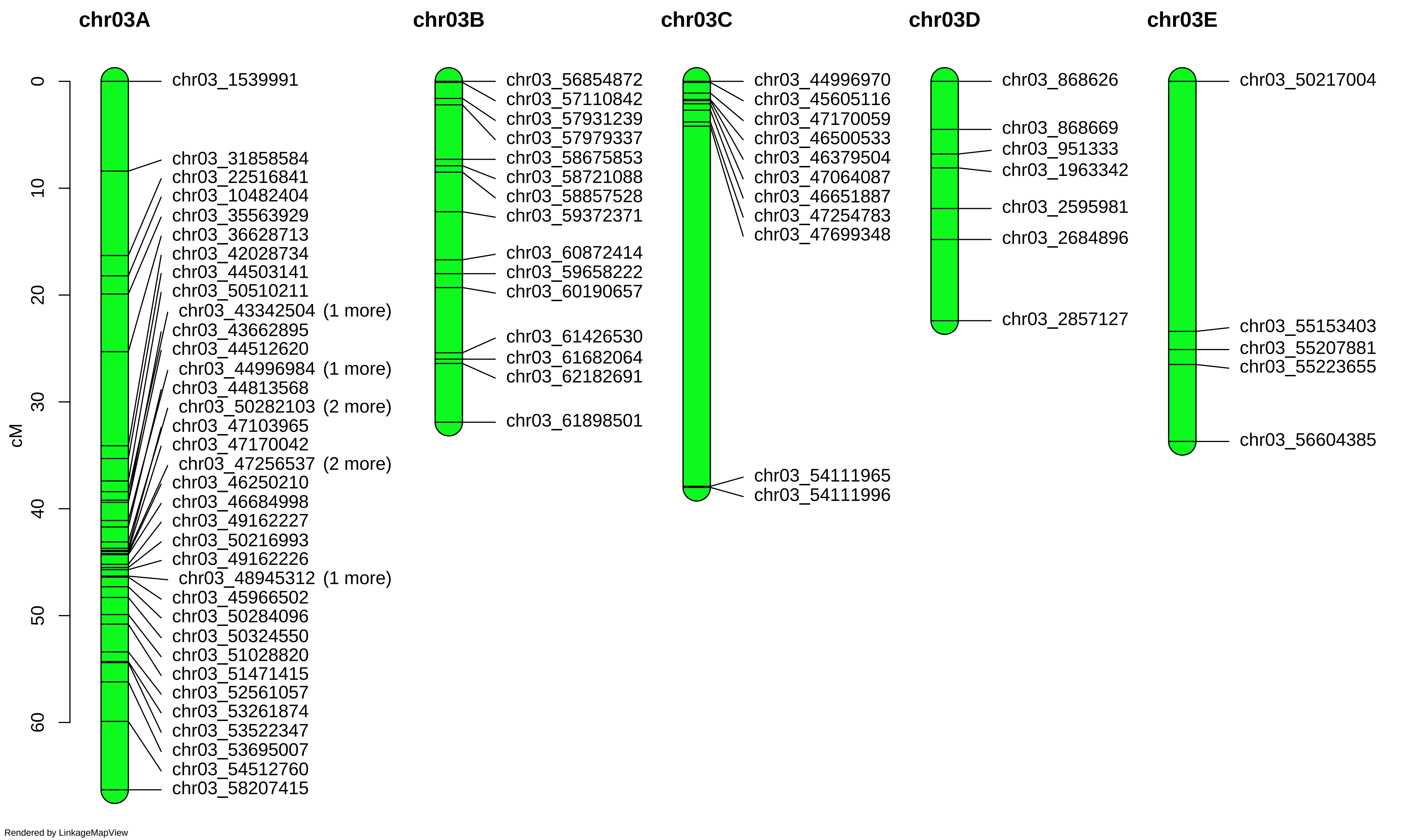
chr03A
chr03B
chr03C
chr03D
chr03E
cM
0
10
20
30
40
50
60
chr03_1539991
chr03_31858584
chr03_22516841
chr03_10482404
chr03_35563929
chr03_36628713
chr03_42028734
chr03_44503141
chr03_50510211
chr03_43342504 (1 more)
chr03_43662895
chr03_44512620
chr03_44996984 (1 more)
chr03_44813568
chr03_50282103 (2 more)
chr03_47103965
chr03_47170042
chr03_47256537 (2 more)
chr03_46250210
chr03_46684998
chr03_49162227
chr03_50216993
chr03_49162226
chr03_48945312 (1 more)
chr03_45966502
chr03_50284096
chr03_50324550
chr03_51028820
chr03_51471415
chr03_52561057
chr03_53261874
chr03_53522347
chr03_53695007
chr03_54512760
chr03_58207415
chr03_56854872
chr03_57110842
chr03_57931239
chr03_57979337
chr03_58675853
chr03_58721088
chr03_58857528
chr03_59372371
chr03_60872414
chr03_59658222
chr03_60190657
chr03_61426530
chr03_61682064
chr03_62182691
chr03_61898501
chr03_44996970
chr03_45605116
chr03_47170059
chr03_46500533
chr03_46379504
chr03_47064087
chr03_46651887
chr03_47254783
chr03_47699348
chr03_54111965
chr03_54111996
chr03_868626
chr03_868669
chr03_951333
chr03_1963342
chr03_2595981
chr03_2684896
chr03_2857127
chr03_50217004
chr03_55153403
chr03_55207881
chr03_55223655
chr03_56604385
Rendered by LinkageMapView

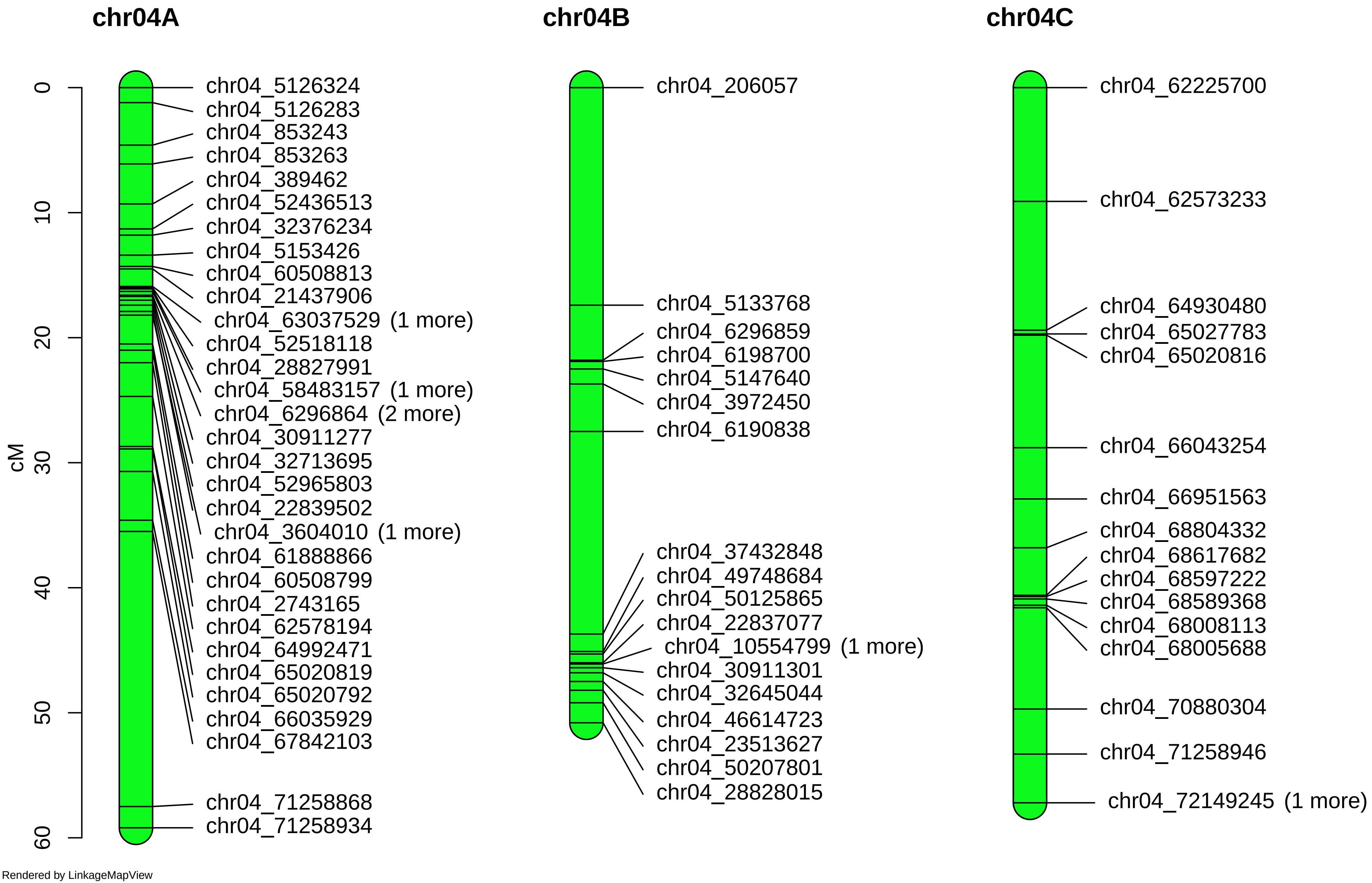
chr04A
chr04B
chr04C
cM
0
10
20
30
40
50
60
chr04_5126324
chr04_5126283
chr04_853243
chr04_853263
chr04_389462
chr04_52436513
chr04_32376234
chr04_5153426
chr04_60508813
chr04_21437906
chr04_63037529 (1 more)
chr04_52518118
chr04_28827991
chr04_58483157 (1 more)
chr04_6296864 (2 more)
chr04_30911277
chr04_32713695
chr04_52965803
chr04_22839502
chr04_3604010 (1 more)
chr04_61888866
chr04_60508799
chr04_2743165
chr04_62578194
chr04_64992471
chr04_65020819
chr04_65020792
chr04_66035929
chr04_67842103
chr04_71258868
chr04_71258934
chr04_206057
chr04_5133768
chr04_6296859
chr04_6198700
chr04_5147640
chr04_3972450
chr04_6190838
chr04_37432848
chr04_49748684
chr04_50125865
chr04_22837077
chr04_10554799 (1 more)
chr04_30911301
chr04_32645044
chr04_46614723
chr04_23513627
chr04_50207801
chr04_28828015
chr04_62225700
chr04_62573233
chr04_64930480
chr04_65027783
chr04_65020816
chr04_66043254
chr04_66951563
chr04_68804332
chr04_68617682
chr04_68597222
chr04_68589368
chr04_68008113
chr04_68005688
chr04_70880304
chr04_71258946
chr04_72149245 (1 more)
Rendered by LinkageMapView

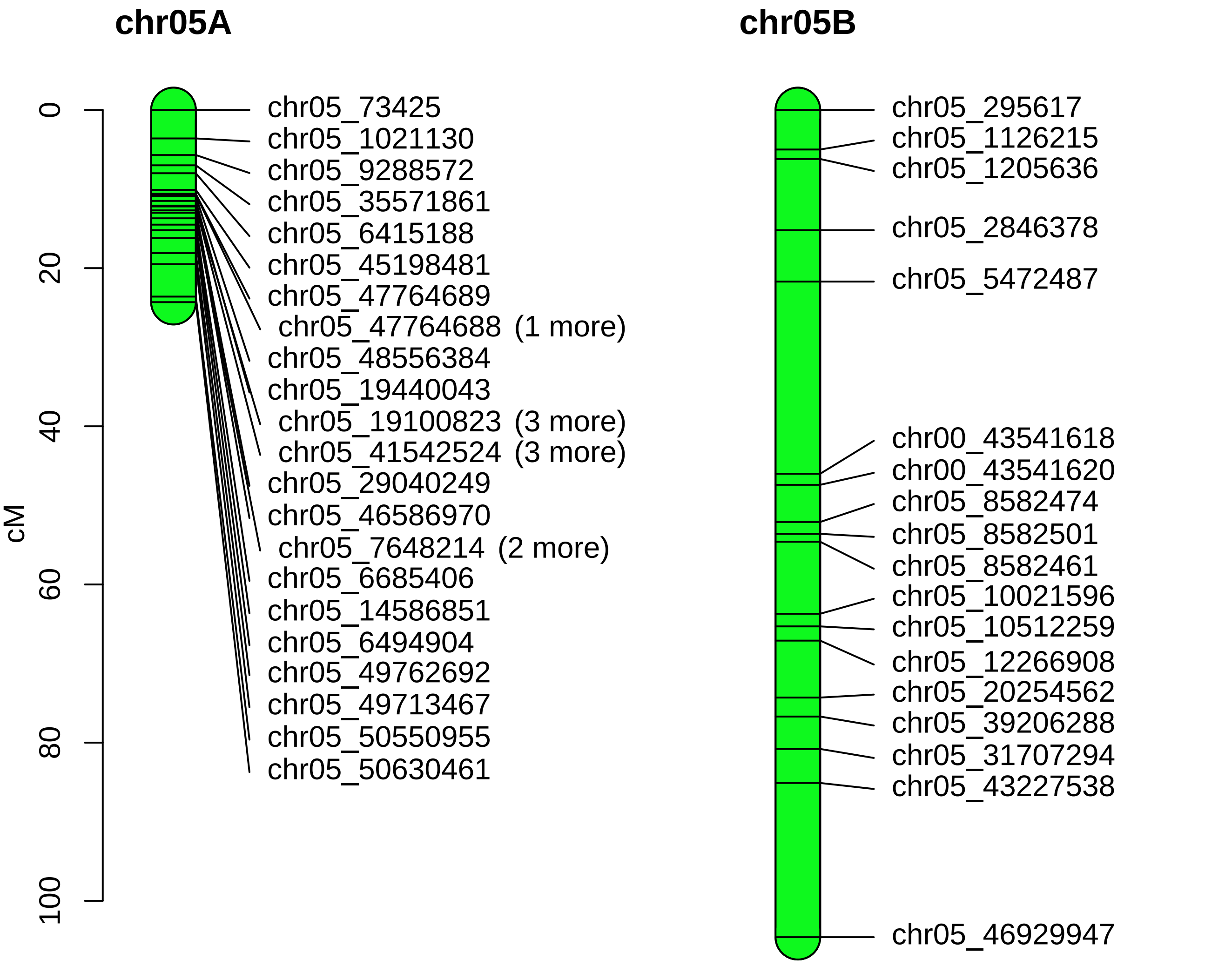

Rendered by LinkageMapView

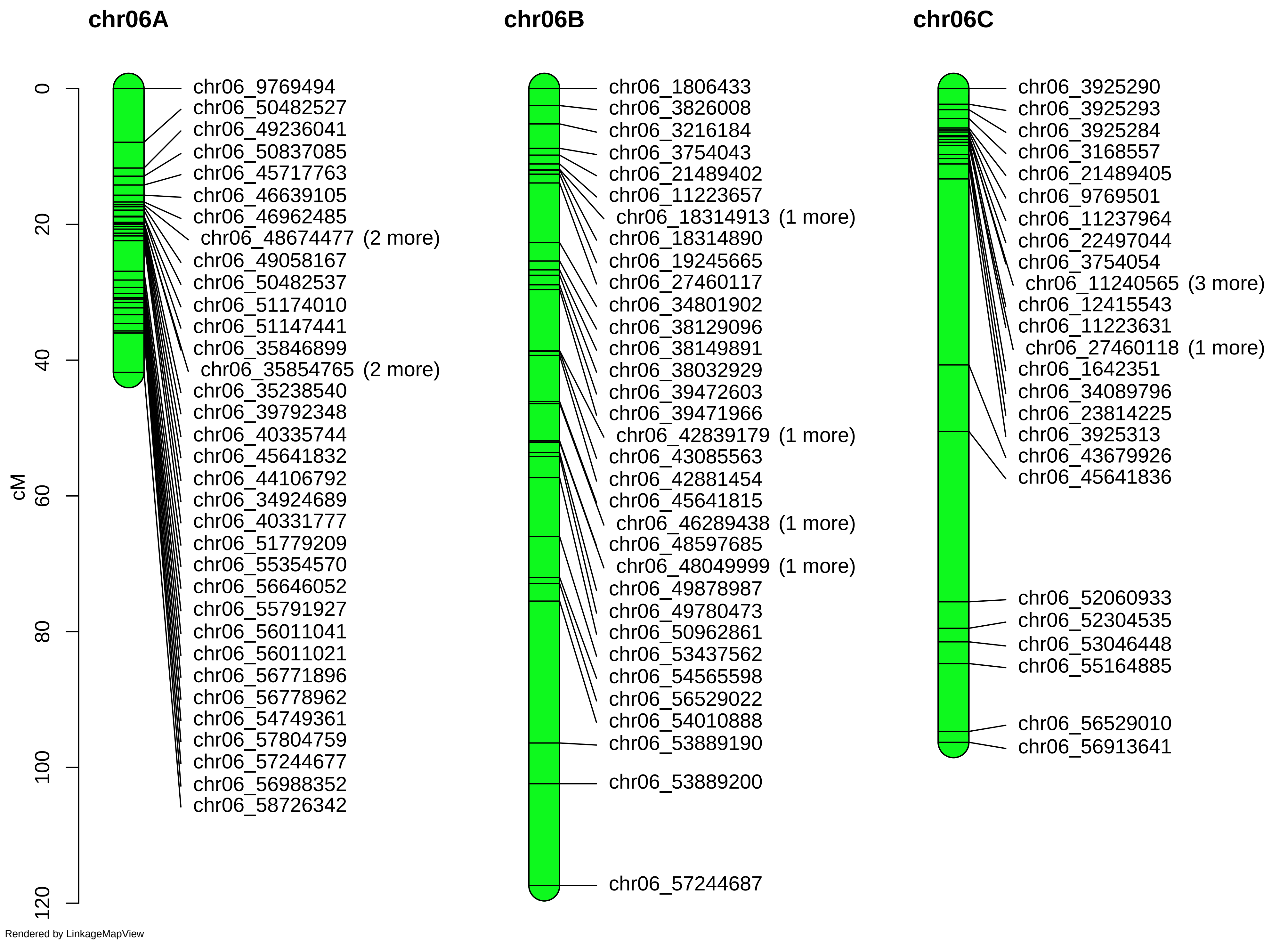
chr06A
chr06B
chr06C
cM
0
20
40
60
80
100
120
chr06_9769494
chr06_50482527
chr06_49236041
chr06_50837085
chr06_45717763
chr06_46639105
chr06_46962485
chr06_48674477 (2 more)
chr06_49058167
chr06_50482537
chr06_51174010
chr06_51147441
chr06_35846899
chr06_35854765 (2 more)
chr06_35238540
chr06_39792348
chr06_40335744
chr06_45641832
chr06_44106792
chr06_34924689
chr06_40331777
chr06_51779209
chr06_55354570
chr06_56646052
chr06_55791927
chr06_56011041
chr06_56011021
chr06_56771896
chr06_56778962
chr06_54749361
chr06_57804759
chr06_57244677
chr06_56988352
chr06_58726342
chr06_1806433
chr06_3826008
chr06_3216184
chr06_3754043
chr06_21489402
chr06_11223657
chr06_18314913 (1 more)
chr06_18314890
chr06_19245665
chr06_27460117
chr06_34801902
chr06_38129096
chr06_38149891
chr06_38032929
chr06_39472603
chr06_39471966
chr06_42839179 (1 more)
chr06_43085563
chr06_42881454
chr06_45641815
chr06_46289438 (1 more)
chr06_48597685
chr06_48049999 (1 more)
chr06_49878987
chr06_49780473
chr06_50962861
chr06_53437562
chr06_54565598
chr06_56529022
chr06_54010888
chr06_53889190
chr06_53889200
chr06_57244687
chr06_3925290
chr06_3925293
chr06_3925284
chr06_3168557
chr06_21489405
chr06_9769501
chr06_11237964
chr06_22497044
chr06_3754054
chr06_11240565 (3 more)
chr06_12415543
chr06_11223631
chr06_27460118 (1 more)
chr06_1642351
chr06_34089796
chr06_23814225
chr06_3925313
chr06_43679926
chr06_45641836
chr06_52060933
chr06_52304535
chr06_53046448
chr06_55164885
chr06_56529010
chr06_56913641
Rendered by LinkageMapView

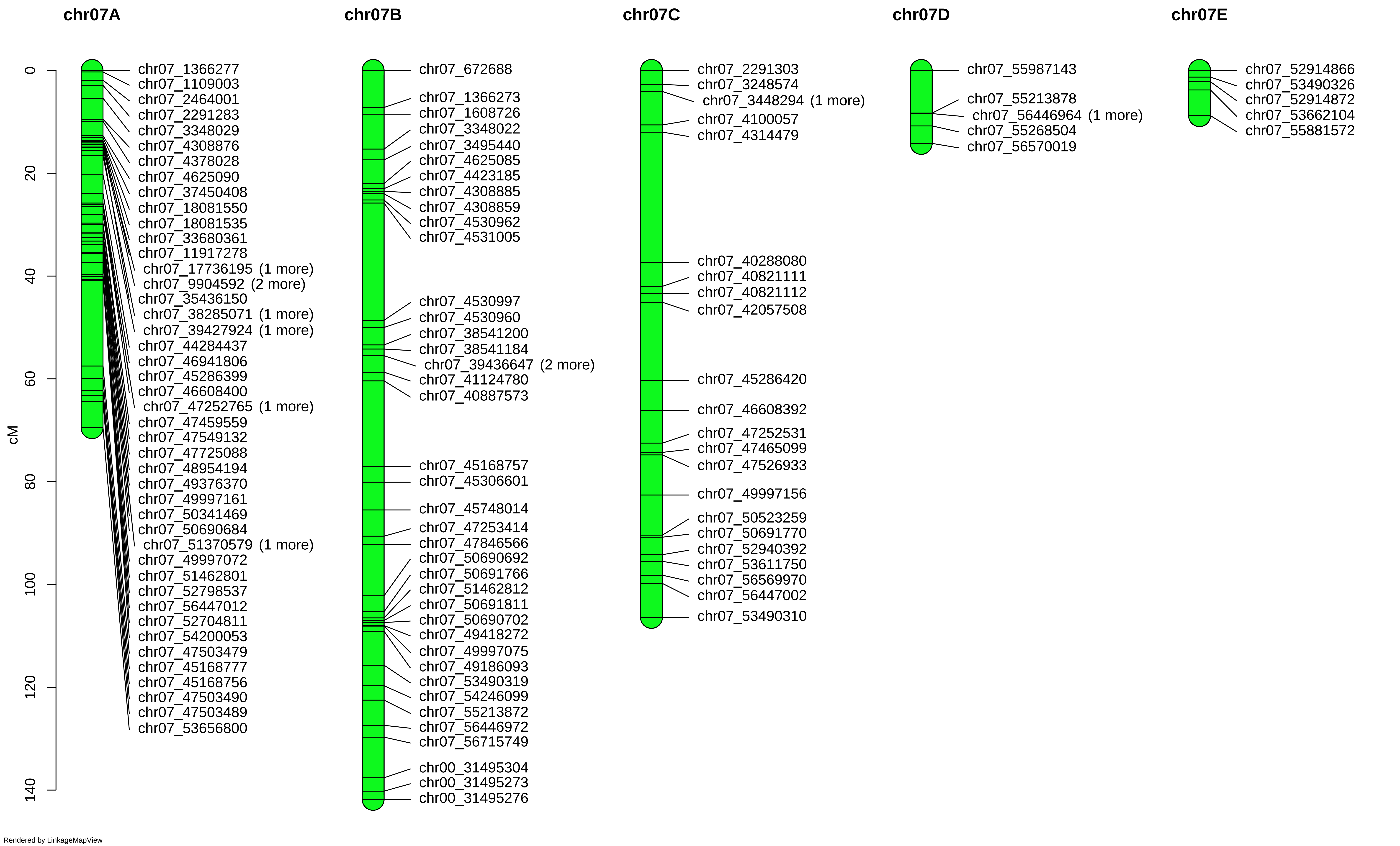
chr07A
chr07B
chr07C
chr07D
chr07E
cM
0
20
40
60
80
100
120
140
chr07_1366277
chr07_1109003
chr07_2464001
chr07_2291283
chr07_3348029
chr07_4308876
chr07_4378028
chr07_4625090
chr07_37450408
chr07_18081550
chr07_18081535
chr07_33680361
chr07_11917278
chr07_17736195 (1 more)
chr07_9904592 (2 more)
chr07_35436150
chr07_38285071 (1 more)
chr07_39427924 (1 more)
chr07_44284437
chr07_46941806
chr07_45286399
chr07_46608400
chr07_47252765 (1 more)
chr07_47459559
chr07_47549132
chr07_47725088
chr07_48954194
chr07_49376370
chr07_49997161
chr07_50341469
chr07_50690684
chr07_51370579 (1 more)
chr07_49997072
chr07_51462801
chr07_52798537
chr07_56447012
chr07_52704811
chr07_54200053
chr07_47503479
chr07_45168777
chr07_45168756
chr07_47503490
chr07_47503489
chr07_53656800
chr07_672688
chr07_1366273
chr07_1608726
chr07_3348022
chr07_3495440
chr07_4625085
chr07_4423185
chr07_4308885
chr07_4308859
chr07_4530962
chr07_4531005
chr07_4530997
chr07_4530960
chr07_38541200
chr07_38541184
chr07_39436647 (2 more)
chr07_41124780
chr07_40887573
chr07_45168757
chr07_45306601
chr07_45748014
chr07_47253414
chr07_47846566
chr07_50690692
chr07_50691766
chr07_51462812
chr07_50691811
chr07_50690702
chr07_49418272
chr07_49997075
chr07_49186093
chr07_53490319
chr07_54246099
chr07_55213872
chr07_56446972
chr07_56715749
chr00_31495304
chr00_31495273
chr00_31495276
chr07_2291303
chr07_3248574
chr07_3448294 (1 more)
chr07_4100057
chr07_4314479
chr07_40288080
chr07_40821111
chr07_40821112
chr07_42057508
chr07_45286420
chr07_46608392
chr07_47252531
chr07_47465099
chr07_47526933
chr07_49997156
chr07_50523259
chr07_50691770
chr07_52940392
chr07_53611750
chr07_56569970
chr07_56447002
chr07_53490310
chr07_55987143
chr07_55213878
chr07_56446964 (1 more)
chr07_55268504
chr07_56570019
chr07_52914866
chr07_53490326
chr07_52914872
chr07_53662104
chr07_55881572
Rendered by LinkageMapView

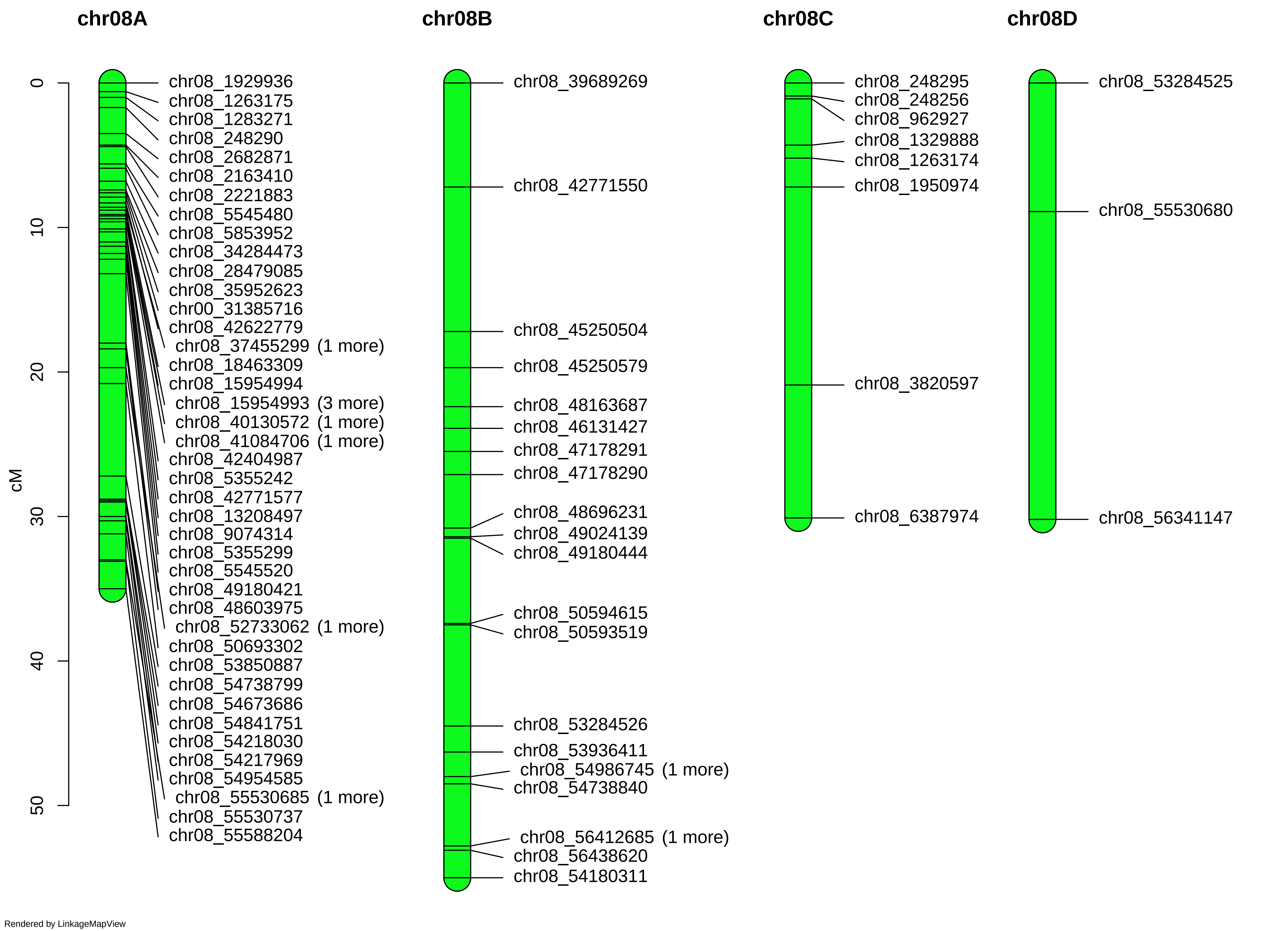
chr08A
chr08B
chr08C
chr08D
cM
0
10
20
30
40
50
chr08_1929936
chr08_1263175
chr08_1283271
chr08_248290
chr08_2682871
chr08_2163410
chr08_2221883
chr08_5545480
chr08_5853952
chr08_34284473
chr08_28479085
chr08_35952623
chr00_31385716
chr08_42622779
chr08_37455299 (1 more)
chr08_18463309
chr08_15954994
chr08_15954993 (3 more)
chr08_40130572 (1 more)
chr08_41084706 (1 more)
chr08_42404987
chr08_5355242
chr08_42771577
chr08_13208497
chr08_9074314
chr08_5355299
chr08_5545520
chr08_49180421
chr08_48603975
chr08_52733062 (1 more)
chr08_50693302
chr08_53850887
chr08_54738799
chr08_54673686
chr08_54841751
chr08_54218030
chr08_54217969
chr08_54954585
chr08_55530685 (1 more)
chr08_55530737
chr08_55588204
chr08_39689269
chr08_42771550
chr08_45250504
chr08_45250579
chr08_48163687
chr08_46131427
chr08_47178291
chr08_47178290
chr08_48696231
chr08_49024139
chr08_49180444
chr08_50594615
chr08_50593519
chr08_53284526
chr08_53936411
chr08_54986745 (1 more)
chr08_54738840
chr08_56412685 (1 more)
chr08_56438620
chr08_54180311
chr08_248295
chr08_248256
chr08_962927
chr08_1329888
chr08_1263174
chr08_1950974
chr08_3820597
chr08_6387974
chr08_53284525
chr08_55530680
chr08_56341147
Rendered by LinkageMapView

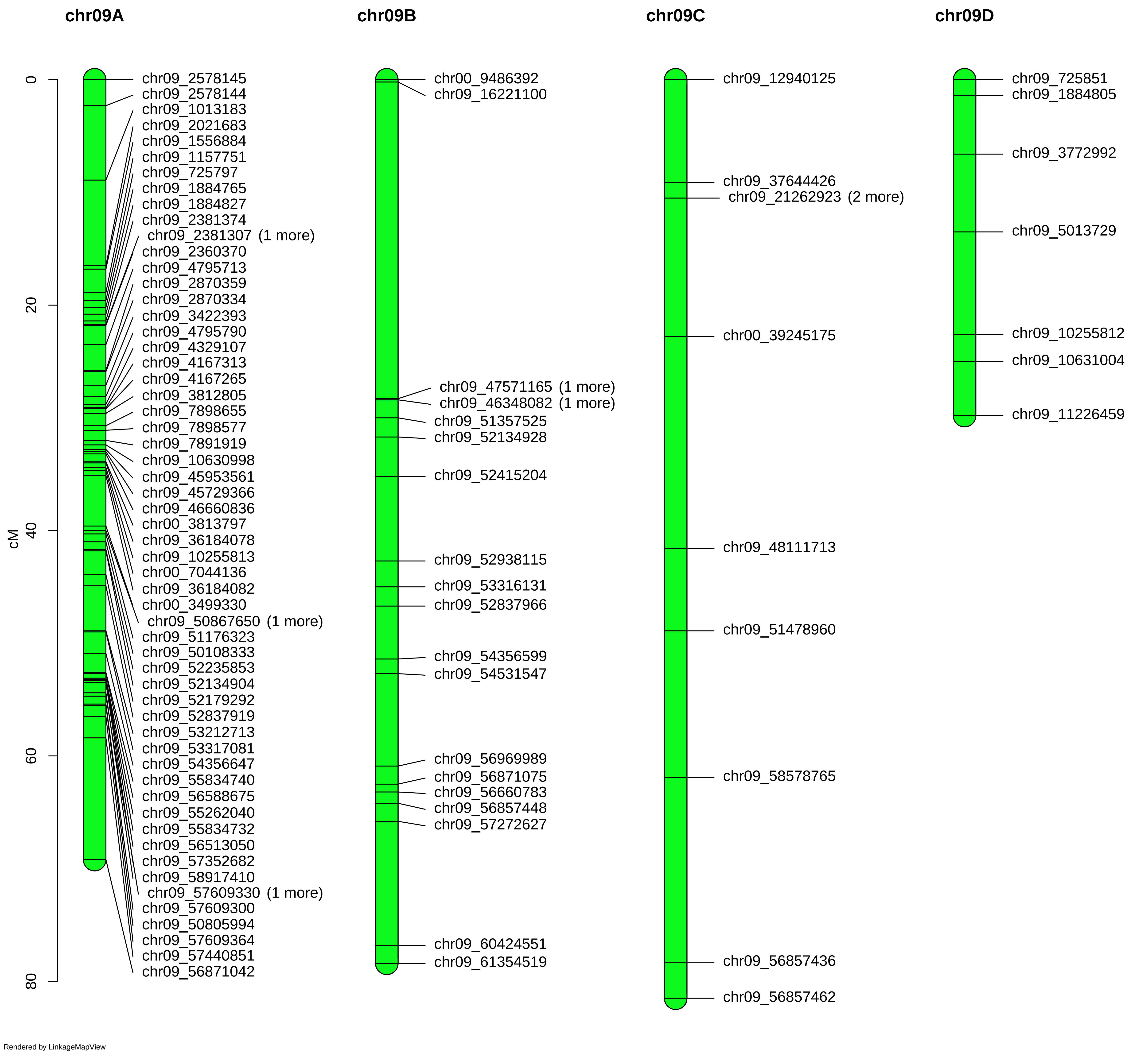

chr09A
chr09B
chr09C
chr09D
cM
0
20
40
60
80
chr09_2578145
chr09_2578144
chr09_1013183
chr09_2021683
chr09_1556884
chr09_1157751
chr09_725797
chr09_1884765
chr09_1884827
chr09_2381374
chr09_2381307 (1 more)
chr09_2360370
chr09_4795713
chr09_2870359
chr09_2870334
chr09_3422393
chr09_4795790
chr09_4329107
chr09_4167313
chr09_4167265
chr09_3812805
chr09_7898655
chr09_7898577
chr09_7891919
chr09_10630998
chr09_45953561
chr09_45729366
chr09_46660836
chr00_3813797
chr09_36184078
chr09_10255813
chr00_7044136
chr09_36184082
chr00_3499330
chr09_50867650 (1 more)
chr09_51176323
chr09_50108333
chr09_52235853
chr09_52134904
chr09_52179292
chr09_52837919
chr09_53212713
chr09_53317081
chr09_54356647
chr09_55834740
chr09_56588675
chr09_55262040
chr09_55834732
chr09_56513050
chr09_57352682
chr09_58917410
chr09_57609330 (1 more)
chr09_57609300
chr09_50805994
chr09_57609364
chr09_57440851
chr09_56871042
chr00_9486392
chr09_16221100
chr09_47571165 (1 more)
chr09_46348082 (1 more)
chr09_51357525
chr09_52134928
chr09_52415204
chr09_52938115
chr09_53316131
chr09_52837966
chr09_54356599
chr09_54531547
chr09_56969989
chr09_56871075
chr09_56660783
chr09_56857448
chr09_57272627
chr09_60424551
chr09_61354519
chr09_12940125
chr09_37644426
chr09_21262923 (2 more)
chr00_39245175
chr09_48111713
chr09_51478960
chr09_58578765
chr09_56857436
chr09_56857462
chr09_725851
chr09_1884805
chr09_3772992
chr09_5013729
chr09_10255812
chr09_10631004
chr09_11226459
Rendered by LinkageMapView

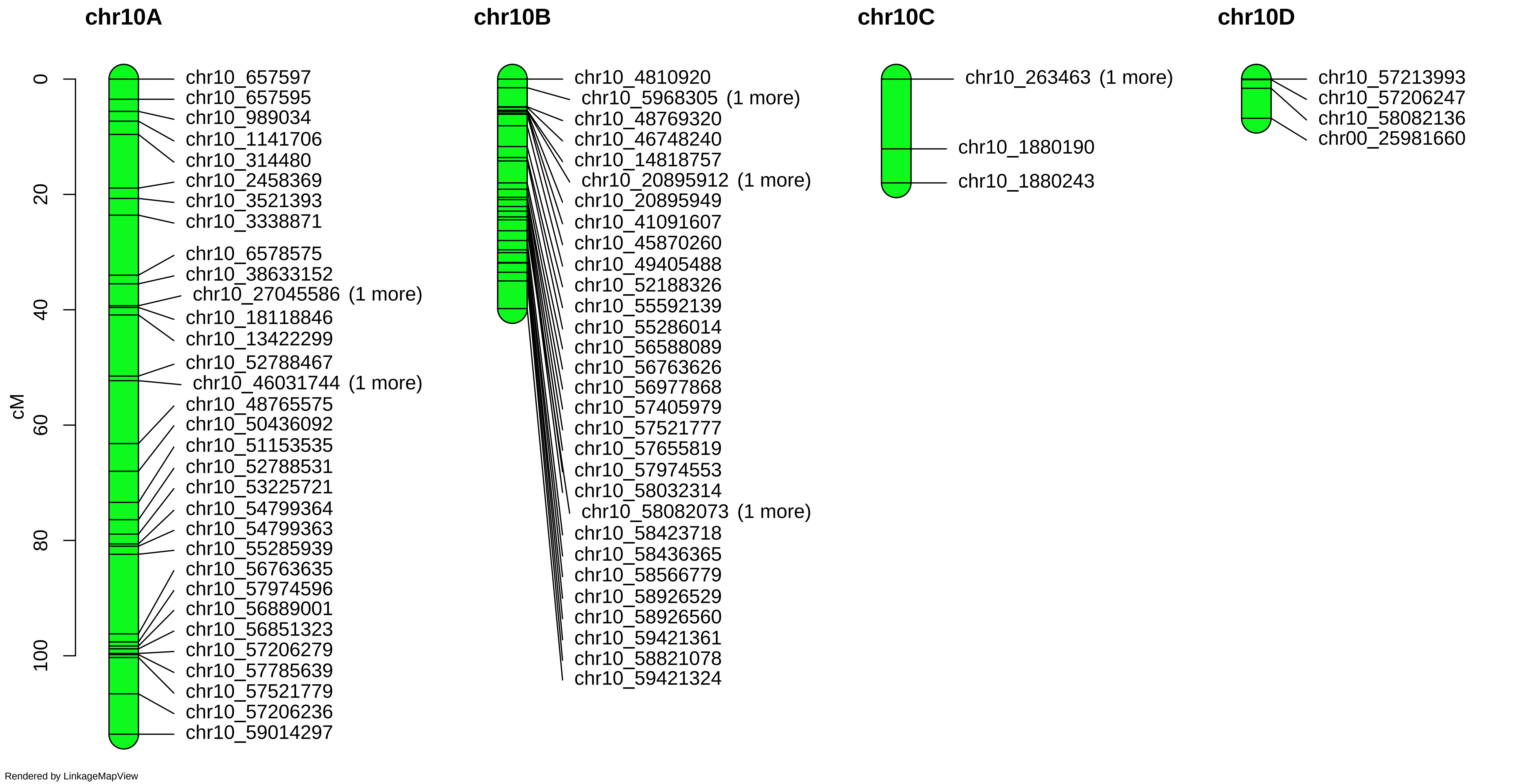
chr10A
chr10B
chr10C
chr10D
cM
0
20
40
60
80
100
chr10_657597
chr10_657595
chr10_989034
chr10_1141706
chr10_314480
chr10_2458369
chr10_3521393
chr10_3338871
chr10_6578575
chr10_38633152
chr10_27045586 (1 more)
chr10_18118846
chr10_13422299
chr10_52788467
chr10_46031744 (1 more)
chr10_48765575
chr10_50436092
chr10_51153535
chr10_52788531
chr10_53225721
chr10_54799364
chr10_54799363
chr10_55285939
chr10_56763635
chr10_57974596
chr10_56889001
chr10_56851323
chr10_57206279
chr10_57785639
chr10_57521779
chr10_57206236
chr10_59014297
chr10_4810920
chr10_5968305 (1 more)
chr10_48769320
chr10_46748240
chr10_14818757
chr10_20895912 (1 more)
chr10_20895949
chr10_41091607
chr10_45870260
chr10_49405488
chr10_52188326
chr10_55592139
chr10_55286014
chr10_56588089
chr10_56763626
chr10_56977868
chr10_57405979
chr10_57521777
chr10_57655819
chr10_57974553
chr10_58032314
chr10_58082073 (1 more)
chr10_58423718
chr10_58436365
chr10_58566779
chr10_58926529
chr10_58926560
chr10_59421361
chr10_58821078
chr10_59421324
chr10_263463 (1 more)
chr10_1880190
chr10_1880243
chr10_57213993
chr10_57206247
chr10_58082136
chr00_25981660
Rendered by LinkageMapView

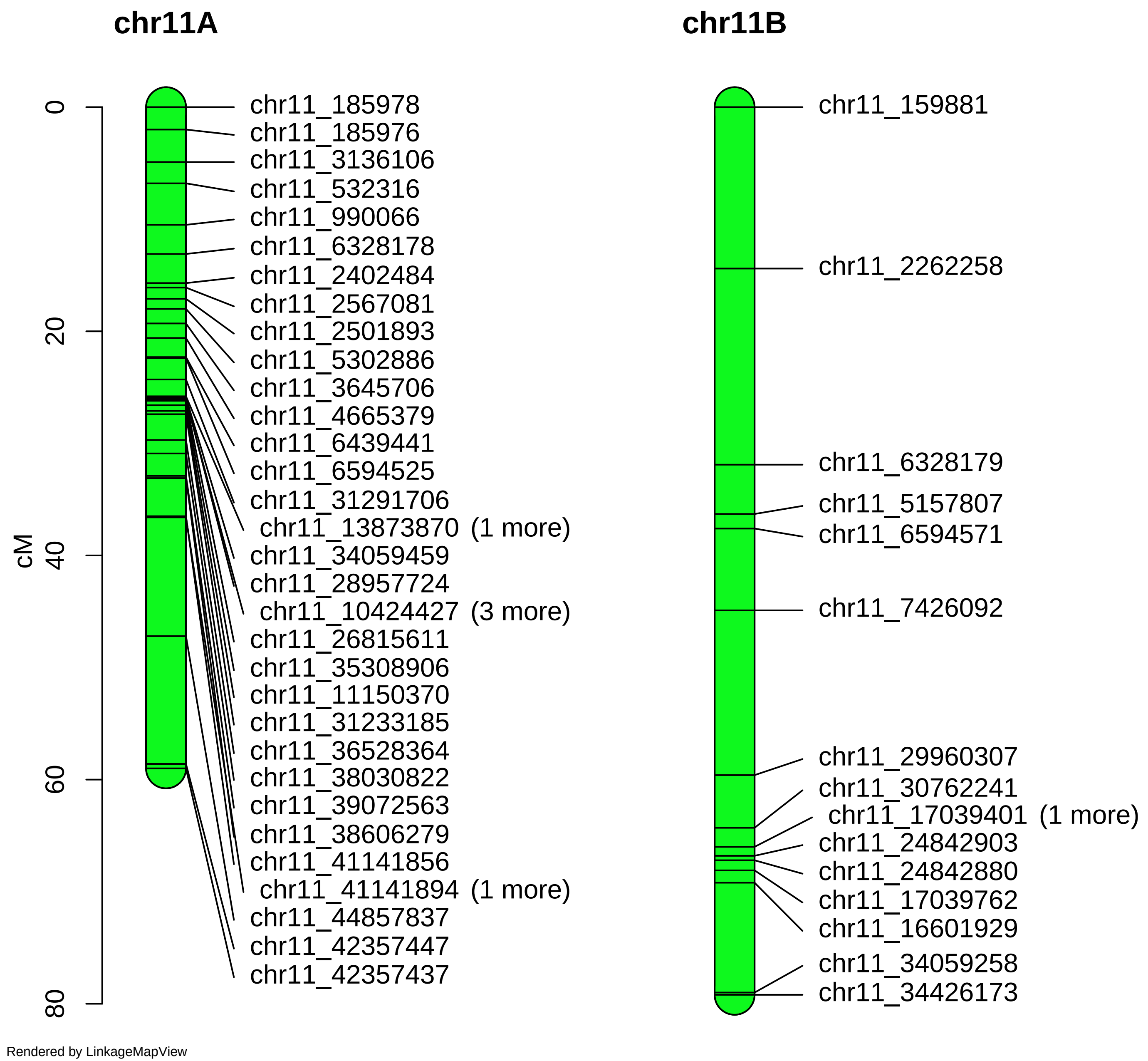
chr11A
chr11B
cM
0
20
40
60
80
chr11_185978
chr11_185976
chr11_3136106
chr11_532316
chr11_990066
chr11_6328178
chr11_2402484
chr11_2567081
chr11_2501893
chr11_5302886
chr11_3645706
chr11_4665379
chr11_6439441
chr11_6594525
chr11_31291706
chr11_13873870 (1 more)
chr11_34059459
chr11_28957724
chr11_10424427 (3 more)
chr11_26815611
chr11_35308906
chr11_11150370
chr11_31233185
chr11_36528364
chr11_38030822
chr11_39072563
chr11_38606279
chr11_41141856
chr11_41141894 (1 more)
chr11_44857837
chr11_42357447
chr11_42357437
chr11_159881
chr11_2262258
chr11_6328179
chr11_5157807
chr11_6594571
chr11_7426092
chr11_29960307
chr11_30762241
chr11_17039401 (1 more)
chr11_24842903
chr11_24842880
chr11_17039762
chr11_16601929
chr11_34059258
chr11_34426173
Rendered by LinkageMapView

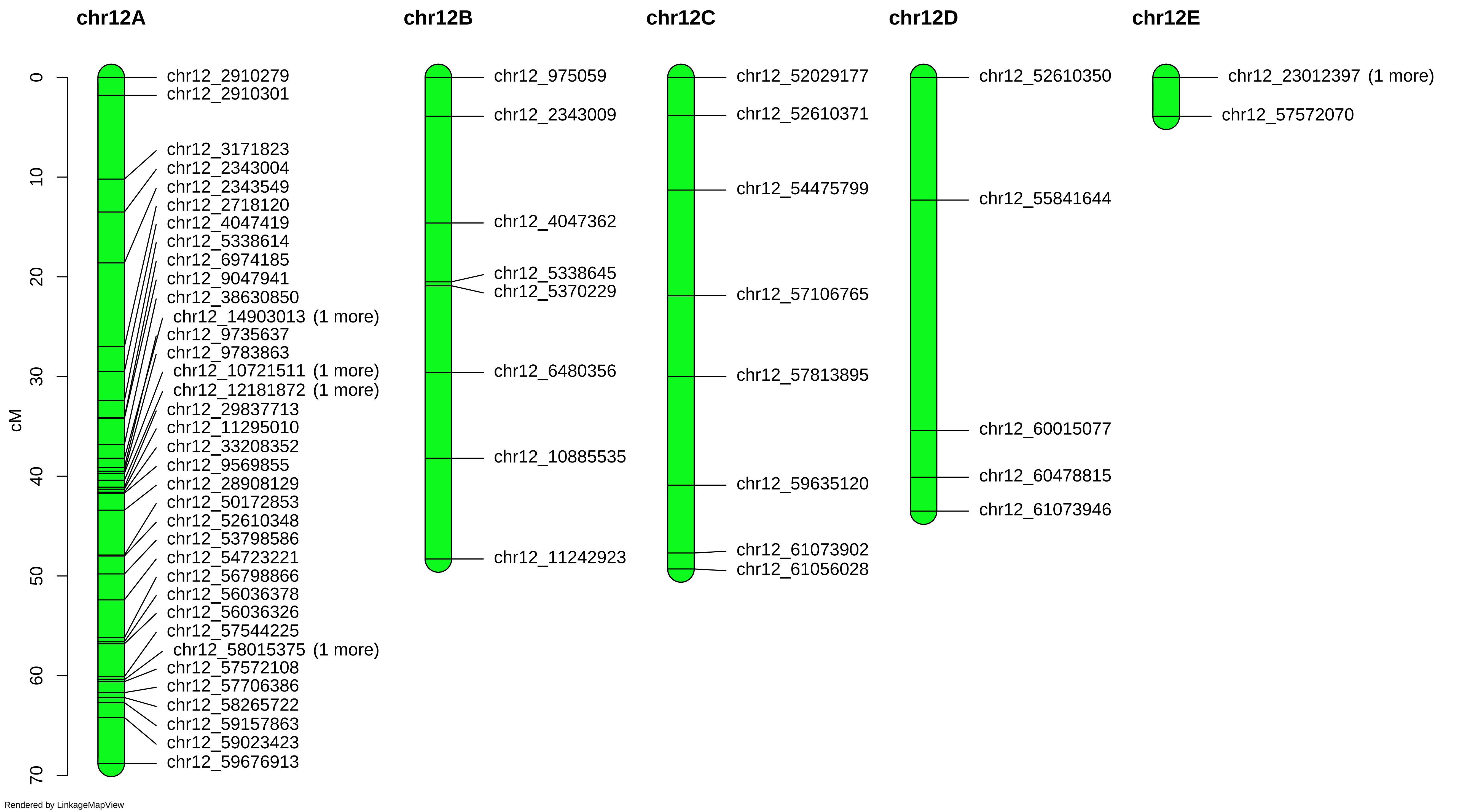
chr12A
chr12B
chr12C
chr12D
chr12E
cM
0
10
20
30
40
50
60
70
chr12_2910279
chr12_2910301
chr12_3171823
chr12_2343004
chr12_2343549
chr12_2718120
chr12_4047419
chr12_5338614
chr12_6974185
chr12_9047941
chr12_38630850
chr12_14903013 (1 more)
chr12_9735637
chr12_9783863
chr12_10721511 (1 more)
chr12_12181872 (1 more)
chr12_29837713
chr12_11295010
chr12_33208352
chr12_9569855
chr12_28908129
chr12_50172853
chr12_52610348
chr12_53798586
chr12_54723221
chr12_56798866
chr12_56036378
chr12_56036326
chr12_57544225
chr12_58015375 (1 more)
chr12_57572108
chr12_57706386
chr12_58265722
chr12_59157863
chr12_59023423
chr12_59676913
chr12_975059
chr12_2343009
chr12_4047362
chr12_5338645
chr12_5370229
chr12_6480356
chr12_10885535
chr12_11242923
chr12_52029177
chr12_52610371
chr12_54475799
chr12_57106765
chr12_57813895
chr12_59635120
chr12_61073902
chr12_61056028
chr12_52610350
chr12_55841644
chr12_60015077
chr12_60478815
chr12_61073946
chr12_23012397 (1 more)
chr12_57572070
Rendered by LinkageMapView

Map	LG	Position (cM)	Locus	chr00 contig	PGSC Pseudomolecule Tiling Path
MCD ALL	chr01	24.237	chr00_13544987	chr00:13530242..13971654	PGSC0003DMB000000464
	chr02	1.339	chr00_21611244	chr00:21579816..21728535	PGSC0003DMB000000756
	chr03	23.829	chr00_40502850	chr00:40502447..40504311	PGSC0003DMB000002378
	chr03	23.986	chr00_8000147	chr00:7515800..8485525	PGSC0003DMB000000254
	chr04	0	chr00_12567719	chr00:12173931..12667864	PGSC0003DMB000000431
	chr04	5.365	chr00_12407838	chr00:12173931..12667864	PGSC0003DMB000000431
	chr04	59.086	chr00_38233239	chr00:38228241..38233666	PGSC0003DMB000001643
	chr08	36.763	chr00_31385668	chr00:31364510..31394384	PGSC0003DMB000001091
	chr10	56.754	chr00_23192845	chr00:23101765..23217759	PGSC0003DMB000000798
	chr11	39.652	chr00_20406095	chr00:20257527..20437853	PGSC0003DMB000000715
MCD MALE	chr01	19.942	chr00_28505932	chr00:28466967..28514885	PGSC0003DMB000000979
	chr02	0.782	chr00_21611244	chr00:21579816..21728535	PGSC0003DMB000000756
	chr03	28.384	chr00_8000147	chr00:7515800..8485525	PGSC0003DMB000000254
	chr04	0	chr00_12407838	chr00:12173931..12667864	PGSC0003DMB000000431
	chr04	58.24	chr00_38233239	chr00:38228241..38233666	PGSC0003DMB000001643
	chr08	11.205	chr00_31385668	chr00:31364510..31394384	PGSC0003DMB000001091
	chr10	61.118	chr00_23192845	chr00:23101765..23217759	PGSC0003DMB000000798
	chr11	52.861	chr00_20406095	chr00:20257527..20437853	PGSC0003DMB000000715
PAM FEMALE	chr01B	51.832	chr00_13962275	chr00:13530242..13971654	PGSC0003DMB000000464
	chr01C	0	chr00_23427320	chr00:23333778..23446661	PGSC0003DMB000000805
	chr02A	61.108	chr00_19558859	chr00:19450431..19661773	PGSC0003DMB000000670
	chr05A	12.092	chr00_37599899	chr00:37597868..37605383	PGSC0003DMB000001545
	chr05B	45.958	chr00_43541618	chr00:43541215..43541950	PGSC0003DMB000004984
	chr05B	47.434	chr00_43541620	chr00:43541215..43541950	PGSC0003DMB000004984
	chr07B	140.205	chr00_31495273	chr00:31483820..31513220	PGSC0003DMB000001096
	chr07B	141.778	chr00_31495276	chr00:31483820..31513220	PGSC0003DMB000001096
	chr07B	137.612	chr00_31495304	chr00:31483820..31513220	PGSC0003DMB000001096
	chr08A	7.861	chr00_31385716	chr00:31364510..31394384	PGSC0003DMB000001091
	chr09A	33.917	chr00_3813797	chr00:1708939..3871400	PGSC0003DMB000000064
	chr09A	34.708	chr00_7044136	chr00:6457363..7515699	PGSC0003DMB000000230
	chr09A	39.603	chr00_3499330	chr00:1708939..3871400	PGSC0003DMB000000064
	chr09B	0	chr00_9486392	chr00:9335487..10101367	PGSC0003DMB000000311
	chr09C	22.756	chr00_39245175	chr00:39242151..39245446	PGSC0003DMB000001879
	chr09C	10.507	chr00_5896866	chr00:6457363..7515699	PGSC0003DMB000000230
	chr09C	10.521	chr00_7044118	chr00:5010086..6318492	PGSC0003DMB000000170
	chr10D	6.82	chr00_25981660	chr00:25967526..26033986	PGSC0003DMB000000896
