# Supplementary material for: Detection of Novel QTLs for Late Blight Resistance Derived from the Wild Potato Species Solanum microdontum and Solanum pampasense
Source: Genes (Basel). 2020 Jun 30;11(7):732. doi: 10.3390/genes11070732 (PMC7396981; doi:10.3390/genes11070732)
Supplement: Supplementary file 1 [file genes-11-00732-s001.zip › Supplementary (revised_)/S2.pdf]

Detection of novel QTLs for late blight resistance derived from the wild potato species *Solanum microdontum* and *Solanum pampasense*

Genes

Fergus Meade, Ronald Hutten, Silke Wagener, Vanessa Prigge, Emmet Dalton, Hanne Grethe Kirk, Denis Griffin, Dan Milbourne

Correspondence: dan.milbourne@teagasc.ie

Trait correlations & additional interval mapping results.

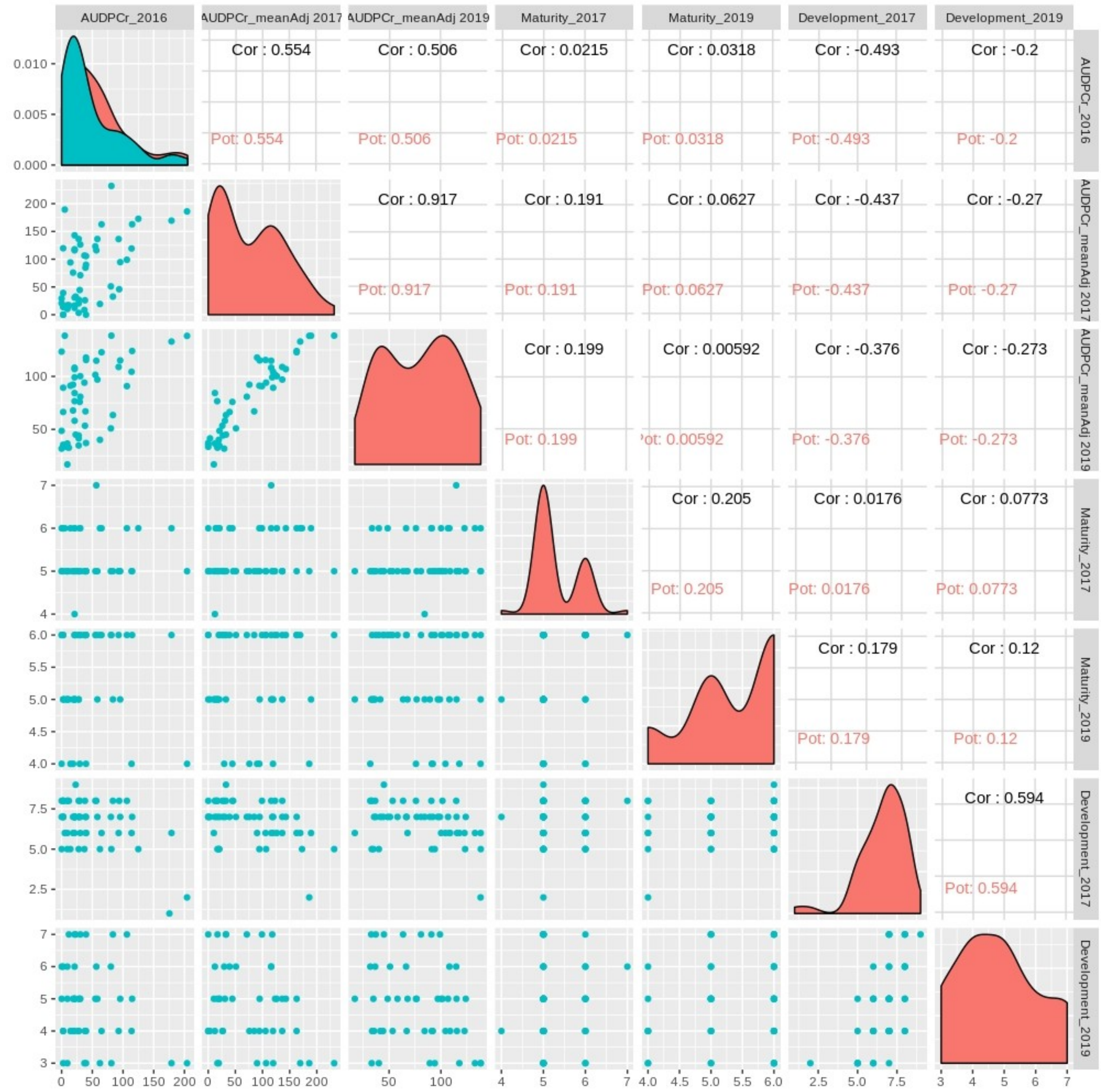

Scatterplots, density plots and correlation calculations for each pair of traits recorded on the MCD population. The "Field subset" of genotypes (blue) was evaluated in 2016 only. Strong correlations in AUDPC were observed between those in the "Pot subset" (red) between years (~0.5 2016 v 2017/2019 & 0.9 2017 v 2019. Development is also negatively correlated with AUDPC in 2017 and 2019.

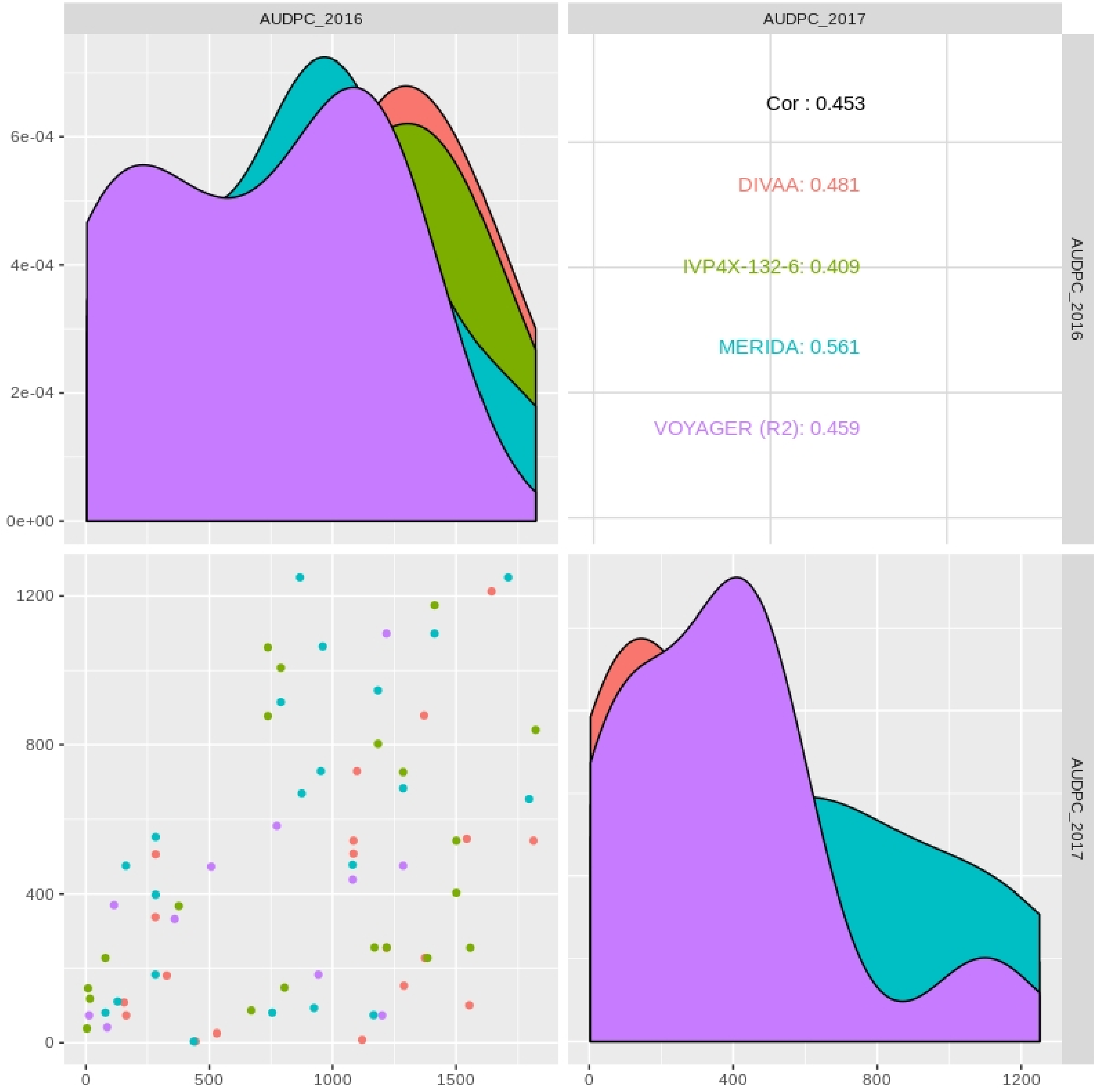

Scatterplot, density plots and correlation calculations for each pair of traits recorded on the PAM population. The colours reflect the male parent in the subpopulation. AUDPC scores were well correlated between years and no one subpopulation appeared to be either resistant or susceptible.

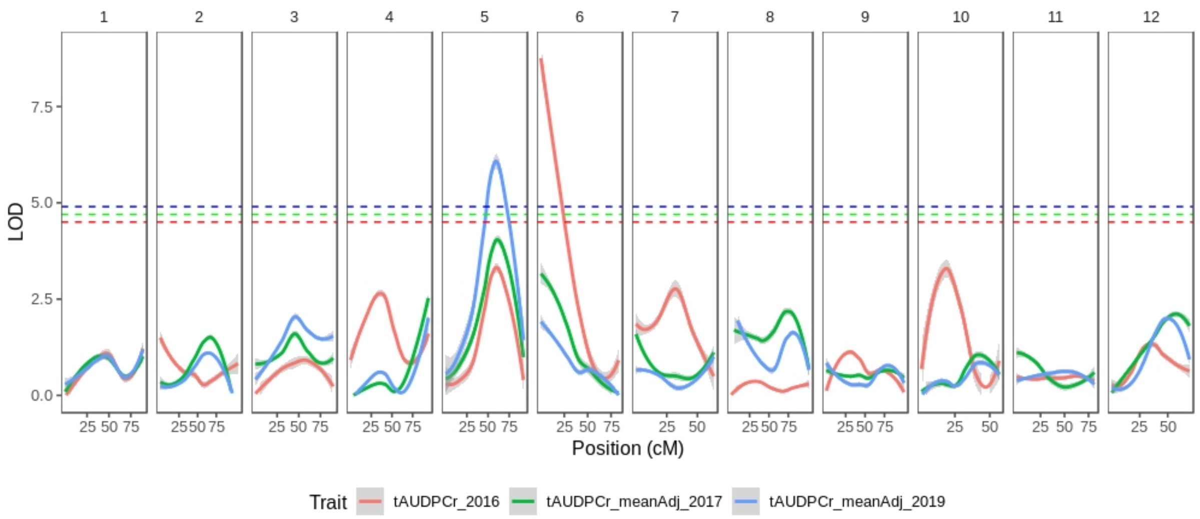

QTL mapping for blight resistance loci using square root transformed AUDPC data. The same loci were identified as with the untransformed phenotypic data.

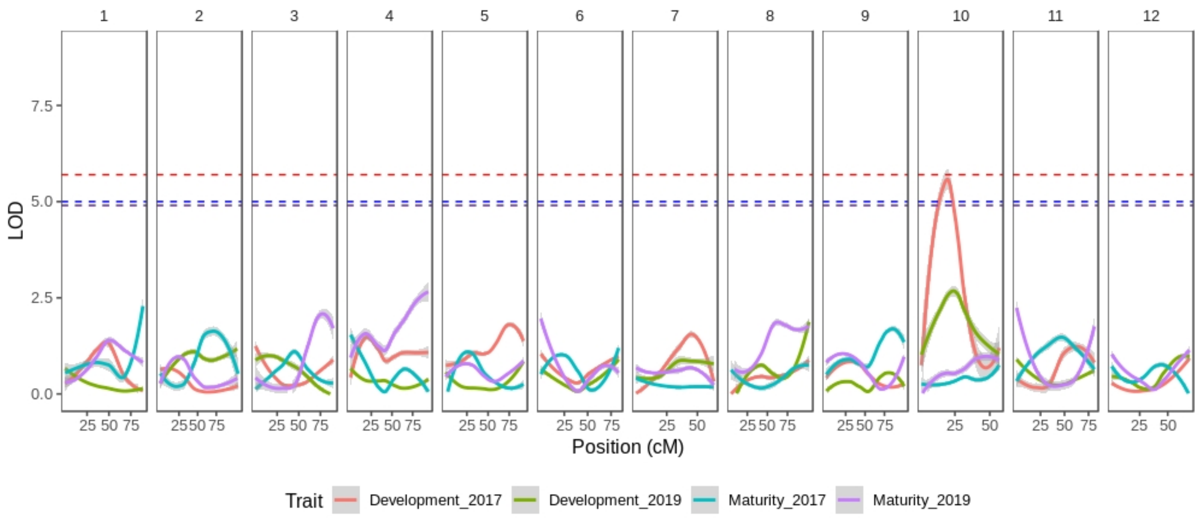

QTL mapping for Maturity and Development traits using the MCD ALL map.

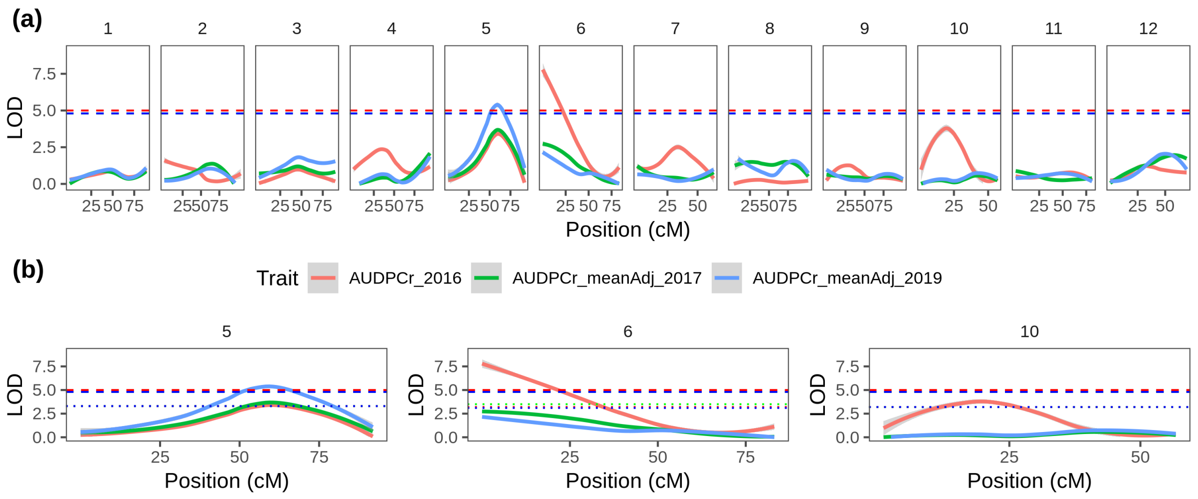

Interval mapping results using the MCD ALL map and each years AUDPC data for a) all linkage groups & b) linkage groups corresponding to chromosomes 5, 6 and 10. Loci ón chr05 and chr06 exceeded the genome wide LOD threshold (dashed lines) in 2019 and 2016, respectively (a). The locus on chr05 exceeded the linkage group specific LOD threshold (dotted lines) each year (b). An inconsistent result was obtained for a locus on chr10 in 2016 only.

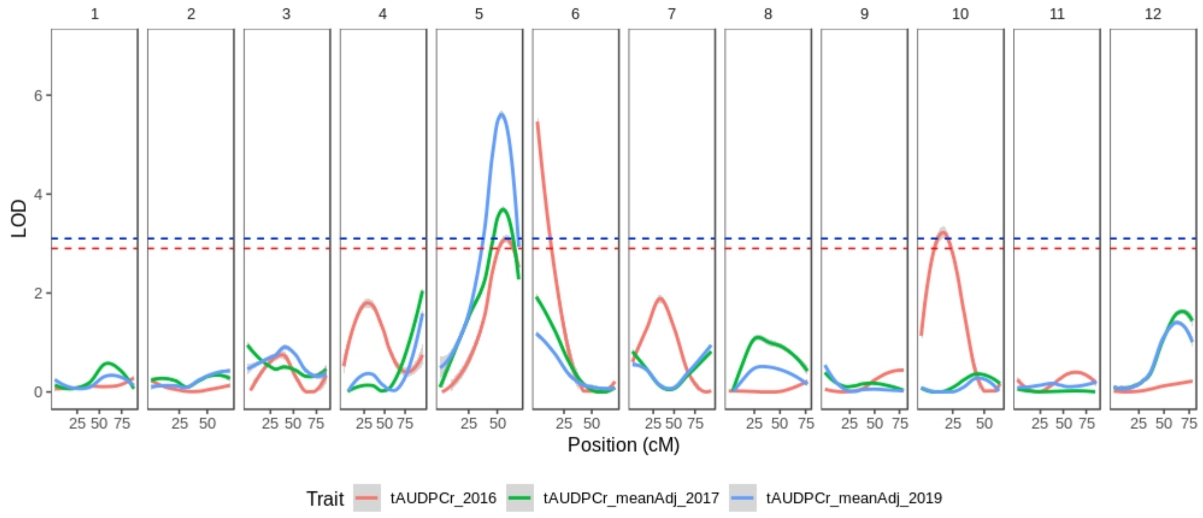

QTL mapping for blight resistance loci using square root transformed AUDPC data and the MCD MALE map. The same loci were identified as with the untransformed phenotypic data.

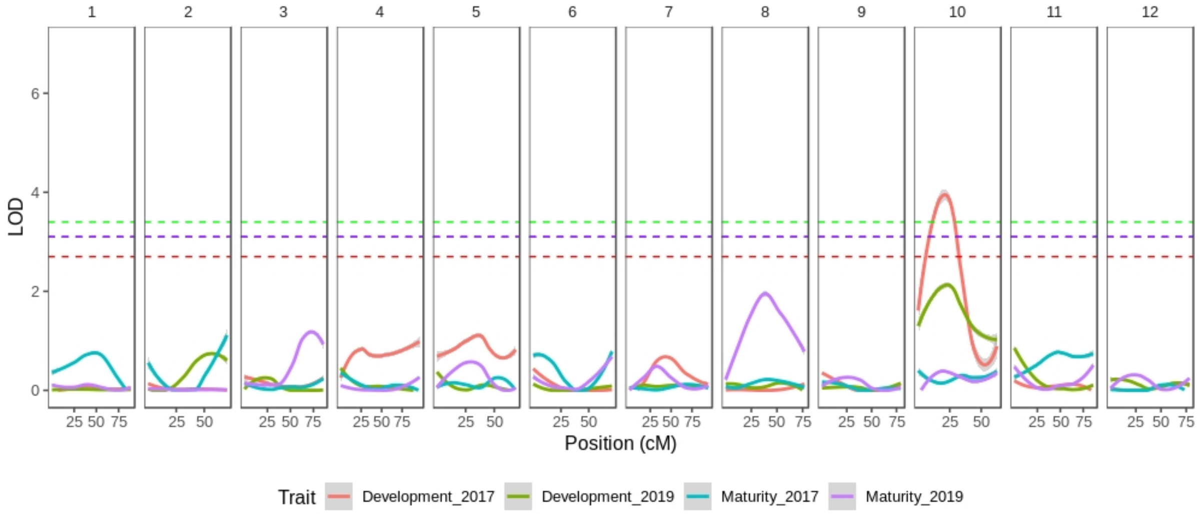

QTL mapping for Development and Maturity traits using the MCD MALE map.

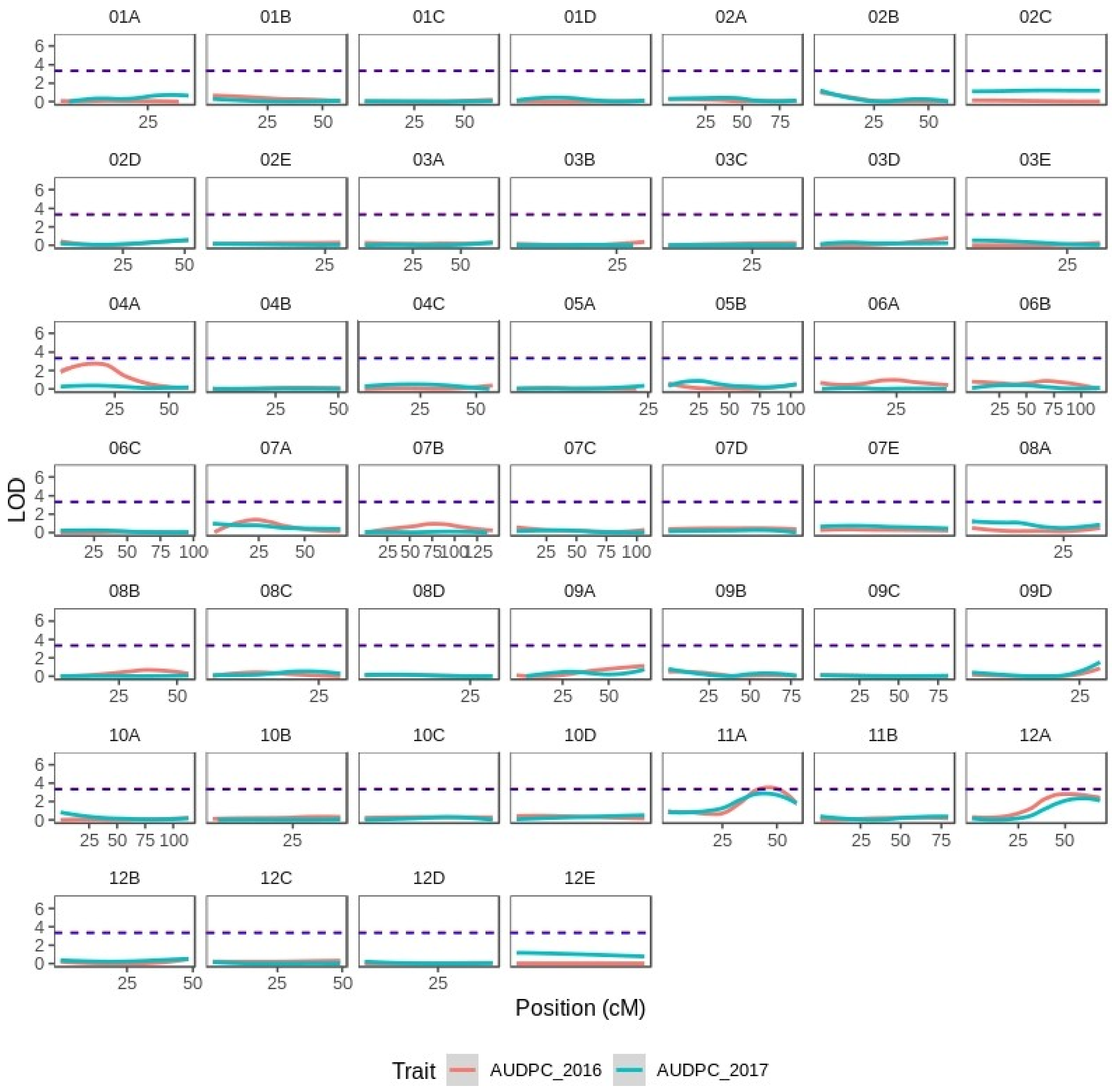

QTL mapping for blight resistance using all 46 linkage groups on the PAM map.

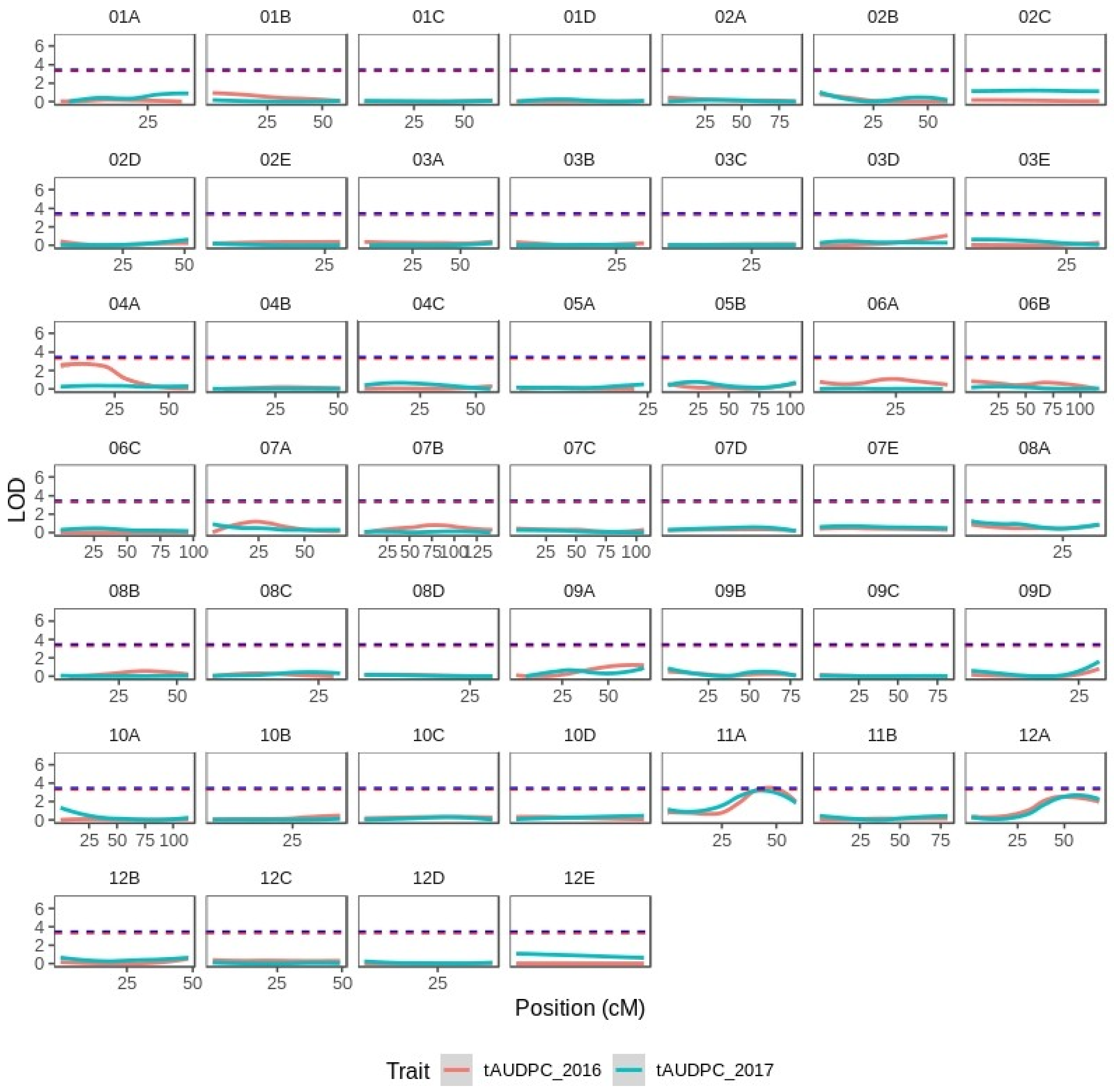

QTL mapping for blight resistance using all 46 linkage groups on the PAM map and square root transformed AUDPC data. The same loci were identified as with the untransformed phenotypic data.
